# Quantifying the impact of biobanks and cohort studies

**DOI:** 10.1073/pnas.2427157122

**Published:** 2025-04-16

**Authors:** Rodrigo Dorantes-Gilardi, Kerry L. Ivey, Lauren Costa, Rachael Matty, Kelly Cho, John Michael Gaziano, Albert-László Barabási

**Affiliations:** ^a^Million Veteran Program Coordinating Center, Veterans Affairs Boston Healthcare System, Boston, MA 02130; ^b^Department of Physics, Network Science Institute, Northeastern University, Boston, MA 02115; ^c^Division of Aging, Department of Medicine, Brigham and Women’s Hospital, Boston, MA 02115; ^d^Department of Medicine, Harvard Medical School, Boston, MA 02115; ^e^Division of General Internal Medicine, Department of Medicine, Brigham and Women’s Hospital, Boston, MA 02115; ^f^Channing Division of Network Medicine, Department of Medicine, Brigham and Women’s Hospital, Harvard Medical School, Boston, MA 02115; ^g^Department of Network and Data Science, Central Eastern University, Budapest 1051, Hungary

**Keywords:** science of science, research impact, biobanks, hidden citations

## Abstract

Biobanks advance biomedical and clinical research by collecting and offering data and biological samples for numerous studies. However, the impact of these repositories varies greatly due to differences in their purpose, scope, governance, and data collected. Here, we computationally identified 2,663 biobanks and their textual mentions in 228,761 scientific articles, 16,210 grants, 15,469 patents, 1,769 clinical trials, and 9,468 public policy documents, helping characterize the academic communities that utilize and support them. We found a strong concentration of biobank-related research on a few diseases, including obesity, Alzheimer’s disease, breast cancer, and diabetes. Moreover, collaboration, rather than citation count, shapes the community’s recognition of a biobank. We show that, on average, 41.1% of articles fail to reference any of the biobank’s reference papers, but 59.6% include a biobank member as a coauthor. Using a generalized linear model, we identified the key factors that contribute to the impact of a biobank, finding that an impactful biobank tends to be more open to external researchers and that quality data—especially linked medical records—as opposed to large data, correlates with a higher impact in science, innovation, and disease. The collected data and findings are accessible through an open-access web application intended to inform strategies to expand access and maximize the value of these resources.

## Significance

Understanding how scientific resources drive discovery is crucial for maximizing research impact and allocating funding effectively. While prior studies have explored factors such as public trust, financing challenges, and participation in biobanks—repositories that have revolutionized biomedical research by providing standardized biological samples and data—we present a comprehensive quantitative analysis of biobanks’ impact and use. Our analysis reveals a concentration on limited disease areas, widespread coauthorship-for-access practices, and systemic undercitation of biobank resources. These findings demonstrate that traditional metrics fail to capture the true value of biobanks and offer a framework for evaluating scientific resources.

In 2009, Time magazine listed biobanks among the ten ideas changing the world (1). Indeed, these repositories of human biological samples and associated data have become fundamental resources for biomedical research, indispensable for understanding the genetic basis of disease and accelerating drug discovery (2–4). Biobanks provide essential cohort data for population studies and genome-wide association studies (GWAS) (5, 6), supporting high-impact research worldwide.

One of the first biobanks, The Framingham Heart Study, was established as a cohort study in 1948 to document the health of 5,209 adult residents from Framingham, Massachusetts, helping define the models still used today for cardiovascular and heart disease risk prediction (7, 8). Equally influential is the relatively new UK Biobank, founded in 2006 to collect into a single resource the genetic information, lifestyle, diet, and medical records of 500,000 adults from the United Kingdom (9). The datasets arising from the UK Biobank are widely used to advance our understanding of the genetic bases of disease, genetic epidemiology, and public health (10–13).

Prior studies have used survey data to explore factors related to biobank impact, from public trust to financing rates, and available data (14–18). Yet, quantifying and understanding the scientific impact of biobanks remains a challenging task, given the significant heterogeneity in their goals, usage policies, and cohort characteristics. As a result, we lack a summary-level understanding of the breadth and the diversity of biobanks and the community using them, nor do we have metrics to capture their multidimensional impact, affecting science, patents, clinical trials, and public health (19–22). The problem is more fundamental: We do not know how many biobanks there are (23, 24), what medical areas they cover (2), who uses them (18, 25), and how their impact is being recognized (26). The last point is particularly concerning given the resource-intensive nature of biobank creation and maintenance.

Here, we fill this gap by relying on big data and the tools of Science of Science (27–30) to identify, catalog, and analyze the usage characteristics of 2,663 biobanks, mapping out 228,761 research publications, 16,210 grants, 15,469 patents, 1,769 clinical trials, and 9,468 public policy documents where these resources are textually mentioned. We use this dataset to track the research footprint of each biobank, offering a quantitative analysis of biobank usage, focus, and impact across multiple dimensions, including research, innovation, public health, and disease. To measure a biobank’s true impact, we introduce the Biobank Impact Factor (bIF), a comprehensive metric that tracks its influence across research, funding, patent applications, clinical trials, public health initiatives, and disease. Our data-driven analysis of biobank impact provides insights into how many biobanks there are, what research areas they cover, who uses them, and how biobanks get recognition.

## The Dataset

Based on the definition of a biobank as a “collection of human biological material linked to relevant personal and health information” (31, 32), our dataset includes resources that provide physical or digital human biological data associated with lifestyle, demographic, or health information, such as cohort studies, cancer registries, and large surveys with biological data, as well as tissue, blood, and brain banks.

To identify the true corpus of biobanks, we integrated 16 biobank catalogs and expanded this list by systematically scanning 141,219,539 research articles for mentions of human biobanks (*SI Appendix*, section 1). We employed natural language processing and network similarity techniques to remove duplicated entries (*SI Appendix*, section 2). Finally, we searched Dimensions database (33) for biobank mentions in the text of 5,040,039 grants, 158,390,184 patents, 801,708 clinical trials, and of 1,783,533 public policy documents.

**Fig. 1.**
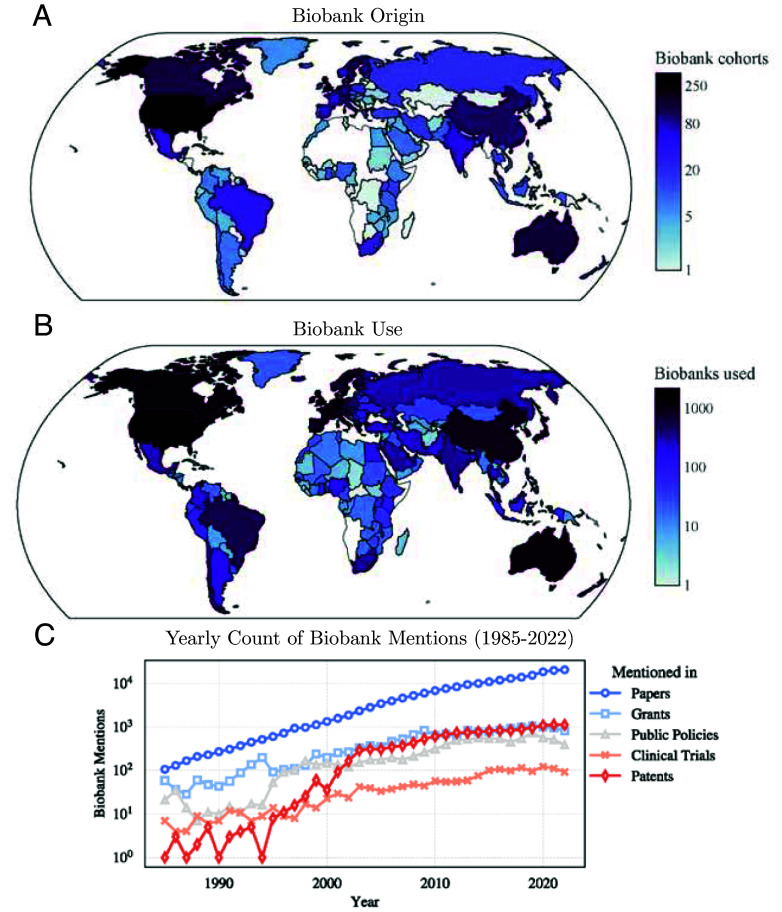
Biobank origin, use, and mentions. (*A*) The origin of biobank cohorts based on the nationalities included in the biobank’s cohort sample (*SI Appendix*, section 9). (*B*) The countries using biobanks based on the affiliation of authors mentioning a biobank in their publications. (*C*) Number of biobank mentions per year across papers, grants, patents, clinical trials, and public policy documents between 1985 and 2022.

Through this computational approach, we identified 2,663 unique biobanks that originated from and were utilized in 139 countries (Fig. 1 *A* and *B*). Collectively, the biobanks were mentioned across 228,761 scientific articles, 16,210 grants, 1,769 clinical trials, 15,469 patents, and 9,468 public policy documents (Fig. 1 *C*). Based on these documents, we extracted additional features related to the biobank’s cohort composition, data offered, and its overall impact (*SI Appendix*, section 9 and Table S1). To allow easy access to the collected data and metrics, we developed an online tool to search, explore, and compare the impact of biobanks, available as a dashboard at http://biobanks.pythonanywhere.com/ and deposited the dataset at https://zenodo.org/records/11671294 (34).

## Results

### The Disease Focus of Biobanks.

**Fig. 2.**
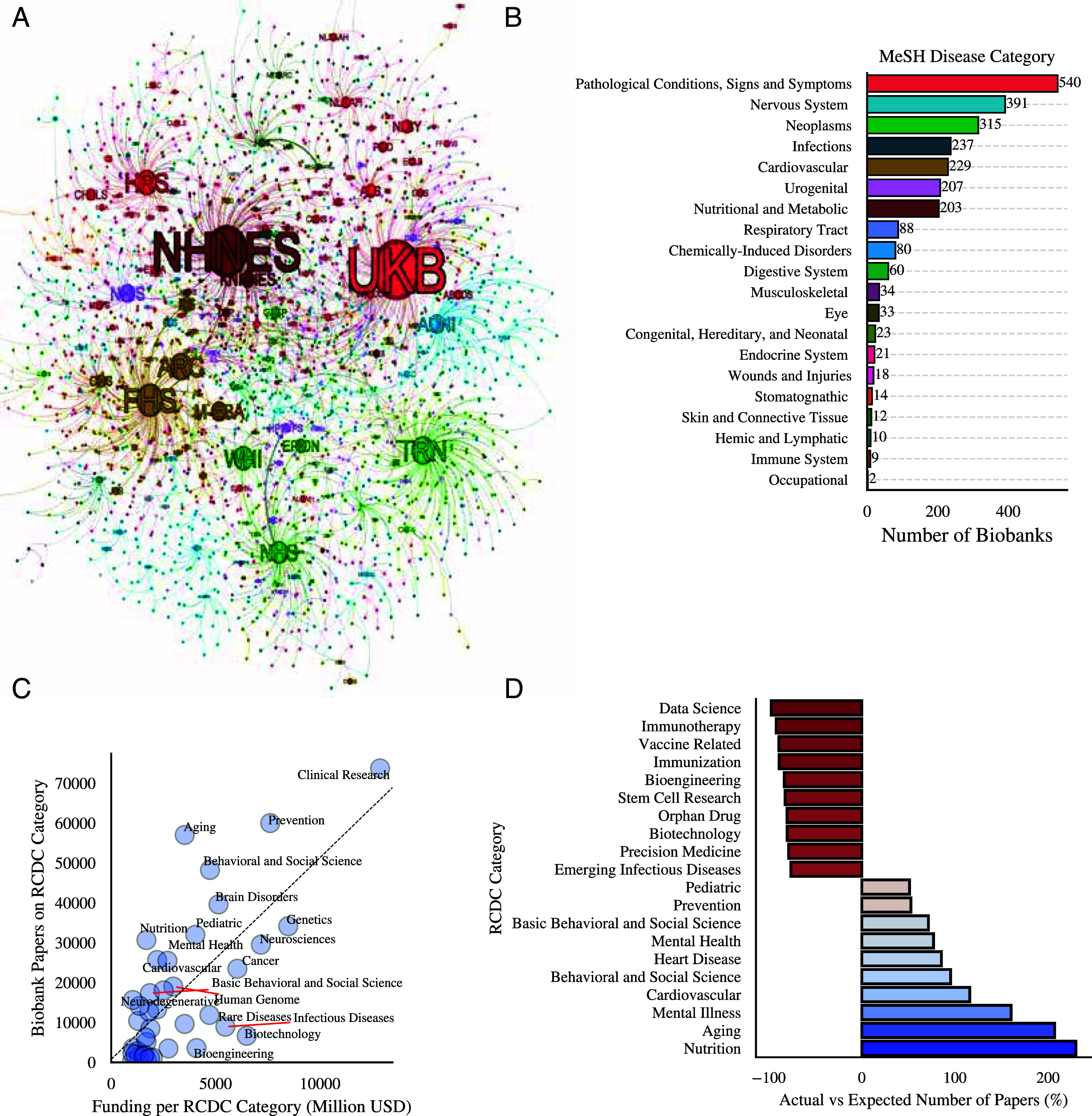
The biobank disease universe. (*A*) The biobank cocitation network whose nodes are biobanks connected by an edge if the same articles frequently cite publications that mention them together. The size of each node is proportional to its number of article mentions, and each node is colored by its principal MeSH disease category (*SI Appendix*, section 7). (*B*) The number of biobanks by disease category representing the communities in the cocitation network. We extracted the Research, Condition, and Disease Categorization (RCDC) classification of biobank publications, along with each RCDC category’s average annual funding by the NIH, to study: (*C*) The relationship between the number of biobank publications and funding per RCDC category. (*D*) Over- and underrepresented RCDC categories in biobank publications measured by the difference between actual and expected publications as a percentage of the number of expected publications on each disease category based on its annual funding.

The most studied diseases by each biobank reflect not only their focus but also the research interests of the scientific community using them. To capture the impact areas of biobanks, we constructed a cocitation network, whose nodes represent individual biobanks, and connections between nodes occur when biobanks’ corresponding publications are cited together (Fig. 2*A* and *SI Appendix*, section 4). We chose cocitations instead of direct citations as they capture the pairwise association of two biobanks through third-party publications rather than a unilateral association. Additionally, we identified the diseases studied by each biobank by analyzing the medical subject headings (MeSH) related to the publications mentioning these resources (*SI Appendix*, section 7.1). From this analysis, we identified 2,901 unique conditions across 20 disease categories based on 111,525 research publications. The network is visibly modular (35), but we find that each community is only partially characterized by the focal disease category of its biobanks, as reflected by their modest normalized mutual information (NMI) score (36) and other overlap metrics (37) (NMI=0.247, *SI Appendix*, section 4.1). This result suggests that biobank communities are formed on more than a single factor, as generally expected for real-world networks (38).

Our analysis shows that biobank research focuses on a few disease categories, with seven of ten biobanks classified as general-purpose, nervous system, urogenital, cancer, infections, or cardiovascular disease (Fig. 2*B*). Combined, these categories account for 80% of all disease-focused publications using biobanks, covering 60% of all studied conditions. Despite finding a similar concentration on few diseases when we look at the 31 million articles with MeSH classifications (*SI Appendix*, section 4.2), we find that cardiovascular (15% of biobank articles vs. 8.7% of total articles) and nutritional diseases (14.3% of biobank articles vs. 4.11% of total articles) are overrepresented in biobank research while investigations on infections (4.4% of biobank articles vs. 12.5% of total articles) and cancer (12.7% of biobank articles vs. 20.8% of total articles) are underrepresented categories. Within each community, we also find a high concentration of research on a few conditions, with obesity, Alzheimer’s disease, breast cancer, and diabetes being studied in one of five publications (*SI Appendix*, Fig. S2). Despite this high concentration across communities and conditions, biobanks demonstrate flexibility in responding to emerging research needs, as captured by the rapid attention to COVID-19 displayed by infectious disease and respiratory tract biobanks.

### Alignment of Funding and Research in Biobank Studies.

To identify the under and overrepresented conditions in biobank research relative to the rest of the biomedical community, we use historical funding data from the NIH to estimate the expected number of publications on each condition. To align with the classification used by the NIH, we extracted the Research, Condition, and Disease Categorization (RCDC) codes of 228,984 biobank publications and associated NIH funding amounts between 2008 and 2022 (*SI Appendix*, section 7.3). Our analysis reveals a strong linear correlation between NIH funding and publication output (r=0.71, $P<10^{-8}$, Fig. 2*C*), suggesting that biobank research increases for categories with a higher available funding. For example, clinical research, the highest-funded category with an annual average of 12.8 billion USD, is the most studied by biobank research, with 73,715 papers. On the other hand, aging is significantly overrepresented in biobank research, ranking third with 57,030 papers, yet only 15th in funding at 3.5 billion USD.

Next, we use the regression’s residuals to evaluate the disparity between a category’s expected and actual research outputs in biobank publications, where a positive (or negative) residual indicates an overrepresentation (or underrepresentation) of the category in biobank research (Fig. 2*D*). We identify 17 RCDC categories overrepresented in biobank research, with an average residual of 11,599 publications per category. Nutrition is the most overrepresented, with over three times the number of expected publications (9,293 expected and 30,673 actual papers, 230% surplus), followed by aging (207% publication surplus), mental illness (160%), and cardiovascular disease (115%). On the other hand, 23 RCDC categories are underrepresented in biobank publications, with an average residual of −8,651 publications. Strongly underrepresented categories include immunization, with less than 12% of the expected number of publications (88% publication deficit), followed by stem cells (82%), orphan drugs (80%), and precision medicine (78%). These results evoke the historically limited focus on a few disorders and their genetic makeup (39), driven potentially by clinical applications rather than commercial interests, as demonstrated by the stronger presence of overrepresented categories in biobank-related clinical trials compared to patents (*SI Appendix*, section 7.3). However, the diversity of biobank research has improved in the last decade, aided significantly by emerging specialized biobanks (40).

### Biobank Impact Factor.

**Fig. 3.**
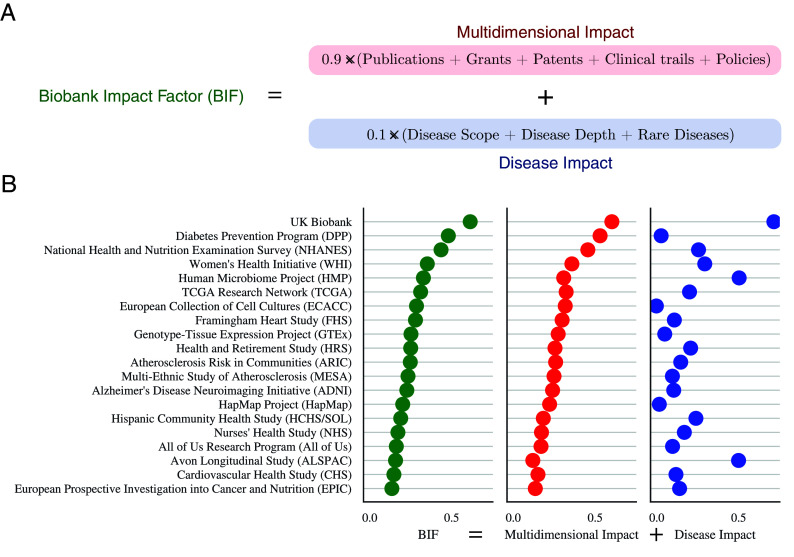
Dimensions of the Biobank Impact Factor (bIF). We built a Biobank Impact Factor based on the number of mentions a biobank has across science, innovation, public policy, and the depth and scope of its disease impact, including rare diseases. (*A*) The formula to calculate bIF based on a weighted sum of the disease impact of a biobank (disease scope and depth and rare disease impact) and its relative number of mentions across each document type (*SI Appendix*, section 10). (*B*) The values of the two metrics composing the bIF are shown for the top 20 biobanks.

While the traditional measure of impact is citation-based, the scientific impact is multifaceted and cannot be fully captured by citations alone (41–43). This is especially true for biobanks, which often lack standardized citation credits (19, 22, 26, 44). Here, we introduce the biobank Impact Factor (Fig. 3*A* and *SI Appendix*, section 10), a metric that integrates multiple dimensions of impact by leveraging the emergence of alternative data sources in biomedicine (45–47) and building on established bioresource evaluation frameworks (44, 48).

To assess the multidimensional impact of a biobank, the bIF combines two key components: research impact (R), and disease impact (D). The research impact quantifies a biobank’s widespread visibility across research publications, grants, patents, clinical trials, and public policies:

[1] $$R=\sum_{i=1}^{5}\frac{r_{i}-\mu_{i}}{\sigma_{i}},$$

where $r_{i}$ represents mentions in research document type i, $\mu_{i}$ and $\sigma_{i}$ are the mean and SD across all biobanks for that document type. To prevent document-specific outliers, each standardized score is normalized at [−1,1], resulting in a total research impact R ranging from −5 to 5, where 5 represents exceptional visibility across all research sectors and −5 indicates minimal presence. On the other hand, the disease impact evaluates a biobank’s contributions across three complementary measures:

[2] $$D=(D_{\text{scope}}+D_{\text{depth}}+D_{\text{rare}})$$

Here, $D_{\text{scope}}$ measures the range of medical conditions studied, $D_{\text{depth}}$ captures the fraction of disease-specific publications mentioning the biobank, and $D_{\text{rare}}$ evaluates impact on rare diseases through publication share (detailed methodology in *SI Appendix*, section 7). Each measure contributes a score between −1 and 1, and their sum D ranges from −3 to 3, where 3 indicates the greatest contributions to all three disease-based metrics and −3 the smallest. The final bIF weights these components to balance broad scientific visibility with disease-specific contributions, normalized by the biobank’s age to ensure fair comparison across biobanks of different ages (see *SI Appendix*, section 10.1 for weight sensitivity analysis):

[3] $$\;\text{bIF}=\frac{1}{Y}\left(0.9\cdot R+0.1\cdot D\right),$$

where Y is the biobank’s age and the resulting bIF varies between −4.8/Y and 4.8/Y, where 4.8 is the maximum impact score in both R and D ((0.9·5+0.1·3)=4.8) and −4.8 the lowest. Hence, a positive bIF indicates above-average impact relative to other biobanks, while a negative bIF suggests below-average performance.

We computed the bIF of all 1,326 biobanks in our dataset with at least 20 publications, a cutoff chosen to have sufficient coverage across disease classes while retaining at least half of the biobanks. Among the biobanks with the highest bIF (Fig. 3*B* and *SI Appendix*, Fig. S4), we find the Diabetes Prevention Program (second, bIF =0.48), the Women’s Health Initiative (fourth, bIF =0.35), the Human Microbiome Project (fifth, bIF =0.32), the Cancer Genome Atlas Program (sixth, bIF =0.31), the Framingham Heart Study (eighth, bIF =0.28), and the Genotype-Tissue Expression Project (ninth, bIF =0.25), all supported by the NIH. The list also includes two UK-based biobanks: The UK Biobank (first, bIF =0.61) and the European Collection of Authenticated Cell Cultures (seventh, bIF =0.29), as well as two other US-based studies, the National Health and Nutrition Examination Survey (third, bIF =0.44), and the Health and Retirement Study (10th, bIF =0.25), completing the top-10 list.

### Biobank Impact is Locally Bounded.

**Fig. 4.**
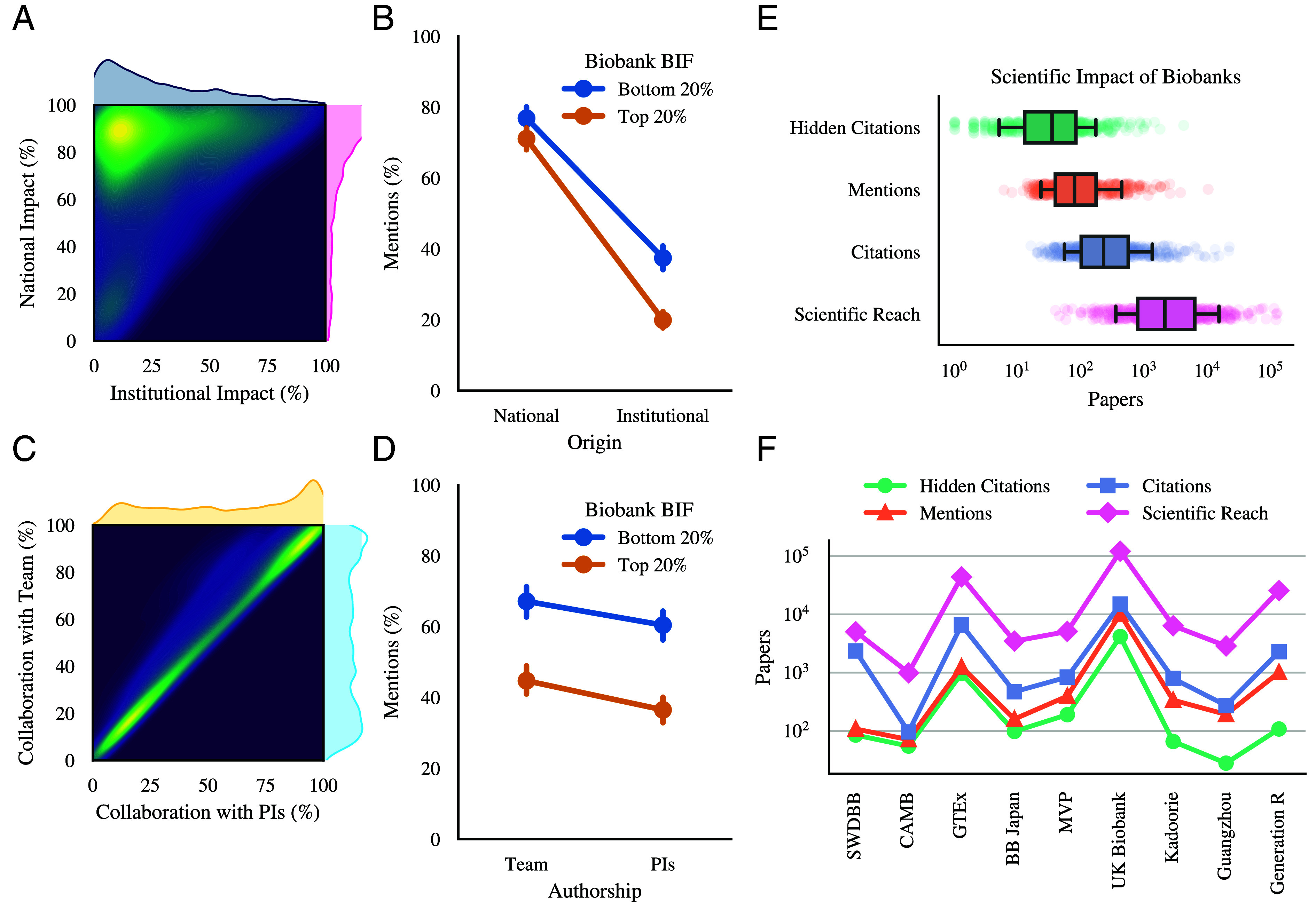
Provenance of biobank research impact and hidden citations. (*A*) The joint distribution of national (same country, purple) and institutional (same research affiliation, pink) impact of biobanks based on mentioning papers. (*B*) Mean percentage and 95% CIs of mentions coming from papers in the same country and institution for bottom-20% (orange) and top-20% biobanks (blue) based on biobank impact factor. Error bars represent 95% CIs. (*C*) Joint distribution of the percentage of mentioning papers listing at least one principal investigator (PI, light yellow) or a team member (light blue) of the biobank. (*D*) Mean percentage and 95% CIs of mentions listing a PI or a biobank team member for bottom-20% (orange) and top-20% (blue) biobanks. (*E*) Distribution of papers mentioning a biobank but not citing its reference papers (green), mentioning a biobank (orange), citing its reference papers (purple), or citing its mentioning papers (biobank reach, pink). Presumably, mentioning articles should include a reference to one of the reference papers of each biobank so the number of hidden citations should be small, as they account for papers for which the biobank is central but fail to cite its main articles. (*F*) Impact metrics for 9 biobanks, including, from left to right: The South West Dementia Brain Bank (SWDBB), Copenhagen Aging and Midlife Biobank (CAMB), Genotype-Tissue Expression (GTEx) Project, BioBank (BB) Japan, Million Veteran Program (MVP), United Kingdom (UK) Biobank, China Kadoorie Biobank, Guangzhou Biobank Cohort Study, and Generation R Study Biobank.

A current survey on biobank use concluded that researchers have a strong preference for local and familiar sources (25), prompting us to measure the extent to which biobanks have local vs. global impact. We first identify the host institution of each biobank (*SI Appendix*, section 5.2) and measure the share of publications mentioning the biobank coming from the host institution or the host country. We find that, on average, 73.5% of the publication impact comes from researchers in the host country of the biobank, and 29.4% have the same institutional affiliation (Fig. 4*A*). We compare these results to a null model where we randomly rewire the citation network while preserving the number of citations of each biobank, finding that the local impact by country and affiliation are highly statistically significant (P-value $<10^{-10}$, *SI Appendix*, section 6).

By comparing biobanks in the top and bottom quintiles of the bIF distribution, we find that while the impact of bottom-20% biobanks is 6% more national than top-20% biobanks (t test P<0.02, Fig. 4*B*), both depend more than 70% on national users, potentially reflecting the challenges of sharing biological data across borders (15, 49). On the other hand, our results show a large and significant difference in institutional impact based on the impact level of a biobank, finding that, on average, institutional impact bottom-20% biobanks is twice as high (37% ± 27%) compared to top-20% biobanks (20% ± 19%, $P<10^{-16}$, Fig. 4*D*). In other words, higher-impact biobanks are, at the same time, less institutional and more international.

### Access to Biobanks Driven by Coauthorship.

Biobanks often restrict scientists’ access to their data, partly driven by privacy and ethical considerations and less justifiably so because maintaining and supplying the data is costly (26). Yet, the often lengthy application process to obtain access to the data is often bypassed via coauthorship with the biobank team, congruent with surveys reporting coauthorship as a prime incentive for biobanks to data sharing (50). This practice has a profound effect on the authorship of the 147,656 articles mentioning a biobank for which we identified its supporting team (*SI Appendix*, section 5.2). Indeed, on average, we find that at least one team member is a coauthor on 59.6% of the articles mentioning the biobank. However, the distribution of the number of coauthorships is bimodal, either very high or almost zero, classifying biobanks into two groups (Fig. 4*C*). Most collaborations resulting in coauthorship occur within the same country (39,496 out of 49,192, 80%) but rarely within the same institution (185, 0.3%), limiting international impact but indicating that the institutional impact of a biobank is purely based on the team’s publications.

Interestingly, the number of coauthorships is markedly different between biobanks at either end of the bIF distribution. Indeed, on average, team members of top-20% biobanks are listed as coauthors in 44% of the papers mentioning the biobank, compared to 67% for bottom-20% biobanks (Fig. 4*D*), suggesting that a lower share of coauthorship of a biobank may be indicative of the demand for the data. Moreover, non-PI members of top-20% biobanks coauthor on average 24 publications without the biobank PIs, a significant percentage (24% of total, P<0.036) compared to bottom-20% biobanks, where non-PIs collaborate in only three papers without the biobank PIs (12%, P=0.06). In other words, collaborative work increases the recognition and scientific impact of biobanks but also sets geographical barriers to their use, limiting their impact.

### Citations Underestimate the True Scientific Impact of Biobanks.

The articles introducing the UK Biobank have been cited 14,995 times (9, 51–54). Yet, we find that 41% of the 10,123 articles mentioning the biobank fail to cite any of them, indicating that many users fail to acknowledge their reliance on the biobank through citation, raising the question, does citation-count capture the true scientific impact of biobanks? We find that not all papers that use biobank data give citation credit to the biobank. Therefore, to estimate the scientific impact of biobanks not visible via citation counts, we identified their “hidden citations”—articles that mention the biobank but fail to cite any of its official publications (55).

To do so, we identified 962 reference papers published by 500 biobanks and evaluated their hidden citations across 96,745 publications (Fig. 4*E* and *SI Appendix*, section 5). Our analysis reveals that, on average, 41.2% of the 203 articles mentioning a biobank fail to cite any of its reference papers, indicating a systemic undercitation of biobanks similar to that observed for software (44, 56). Some strongly undercited biobanks include the GTEx Project (Fig. 4*F*, 967 hidden citations, 77.1% of mentions), the South West Dementia Brain Bank (85 hidden citations, 78.7% of mentions), and the Copenhagen Aging and Midlife Biobank (55 hidden citations, 77.4% of users). Biobanks with a lower number of hidden citations include the Guangzhou Biobank (28, 14.5%), the China Kadoorie Biobank (66, 19.5%), and the Generation R Study (108, 10.6%), indicating that traditional measures of impact, i.e. citations, highly underestimate the true academic impact of biobanks.

Scientific reach, measured by the number of articles citing the publications mentioning a biobank, is a metric used to predict future impact (42). We calculate the reach of each biobank representing its longest stretch of influence, obtaining 120,551 unique papers for the UK Biobank or more than seven times its current detectable citations. On average, we find that the scientific reach of a biobank is 13 times greater than the number of citations (Fig. 4*E*). Note that while these numbers may still underestimate the true impact of biobanks, as we have not scanned the full text of scientific papers, our focus on mentions in titles, abstracts, and acknowledgments helps minimize potential overestimation by excluding casual references that might appear in the main text (*SI Appendix*, section 5.3). This methodological choice provides a more conservative estimate of biobank usage, though it may miss some legitimate uses only mentioned in the paper’s body.

### Biobank Features and their Relation to bIF.

Understanding which variables play a more defining role in their adoption can help biobank creators identify and implement strategies to increase their impact. To differentiate the role of those variables, we designed a generalized linear model explaining the bIF of a biobank (Y), given its set of characteristics (Model **4**).

[4] $$\log\left(Y+1\right)\propto \beta_{0}+\beta_{1}\times X_{1}+\beta_{2}\times X_{2}+\cdots +\beta_{14}\times X_{14}+\epsilon .$$

On the r.h.s we list the 14 features of biobanks that could affect bIF, namely sample size, open data index (*SI Appendix*, section 9.9), PI’s prestige, population or hospital-based, genetic data (gene markers, GWAS, whole-genome sequencing, and gene–environment data), registries, surveys, follow-up data, and medical records. The error term ϵ follows a standard normal distribution. The model was fitted using data from 468 biobanks ($R^{2}=0.41$), and the P-values of the coefficients were Bonferroni corrected. The model’s deviance (0.686) and a Pearson chi-square (0.687) suggest a good fit to the data (*SI Appendix*, section 11).

**Fig. 5.**
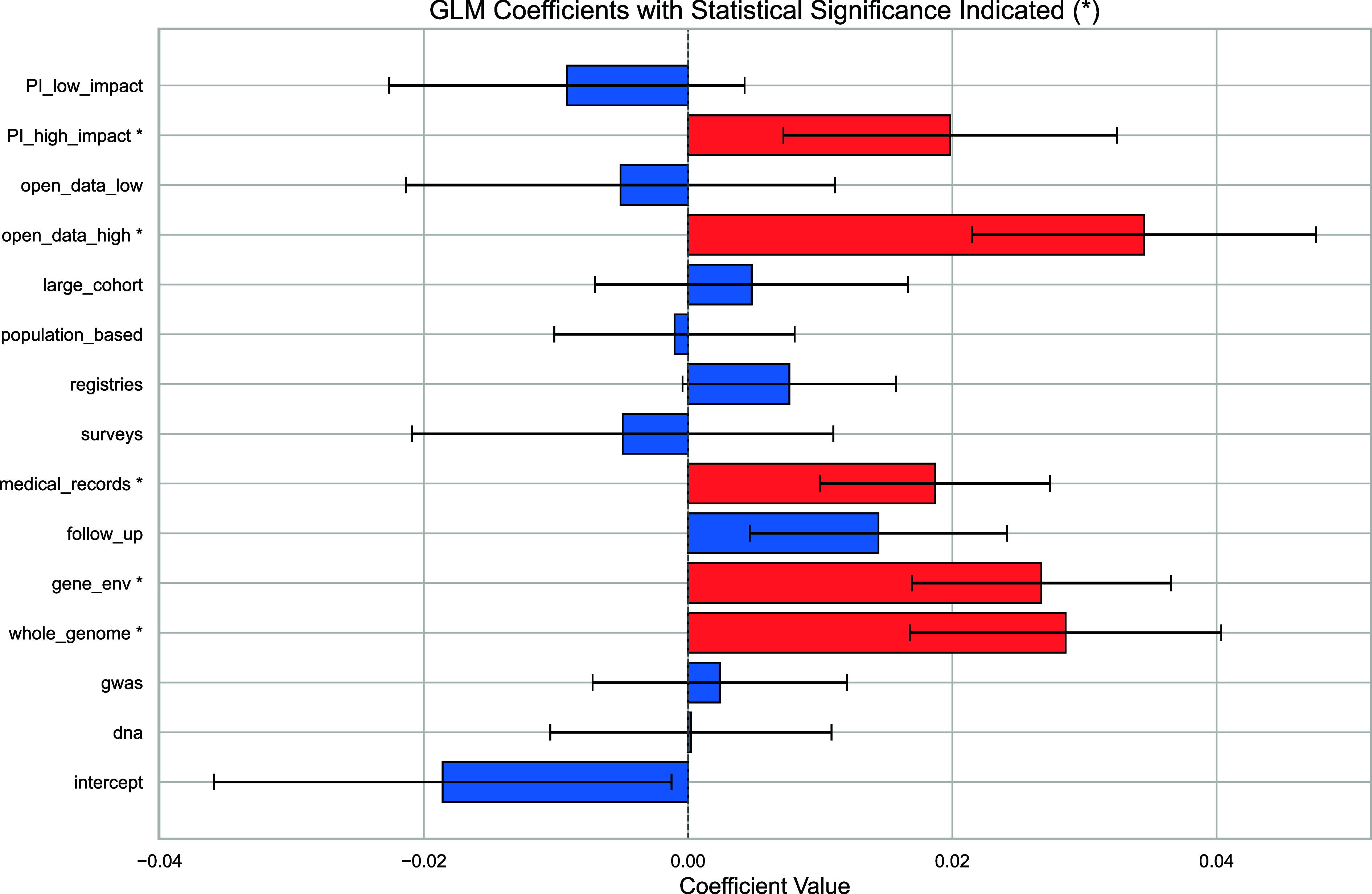
Related biobank features to biobank impact factor. We present a generalized linear model to identify the key features explaining biobank impact. The coefficients of different features are captured by Model **4** considering different binary characteristics of biobanks, including whether the cohort size is large (top 10%), sampled from a general population, data access is open to external researchers, the average citation count of the biobank PIs (bottom and top-10% with respect to all PIs), along with availability of genetic data (subdivided into genetic markers or DNA, GWAS, whole-genome sequencing, or gene–environment interactions), follow-up data, disease-specific data (based on registries), surveys and questionnaires, and linked medical records. Each feature’s coefficient is shown together with its 95% CI. Significant features after applying Bonferroni correction are indicated with a star symbol and red color.

We find five statistically significant coefficients, capturing the more important characteristics related to bIF (Fig. 5). The largest significant coefficient is the one related to a high open-data index ($\beta_{\text{oa}}=0.0345$, P=0.002), followed by whole genome sequencing data ($\beta_{\text{wg}}=0.0286$, $P=2\times 10^{-6}$), gene–environment interaction data ($\beta_{\text{ge}}=0.0267$, $P=9\times 10^{-8}$), a highly cited founder ($\beta_{\text{pi}}=0.0198$, P=0.002), and access to medical records ($\beta_{\text{mr}}=0.0187$, $P=2\times 10^{-5}$). On the other hand, most data features are not significantly related to bIF, including follow-up data (P=0.0037, not significant after Bonferroni correction), surveys (P=0.5), registries (P=0.06), and a large cohort sample (P=0.426). Similarly, not all genetic data help biobank impact, including DNA genetic markers (P=0.96) and GWAS data (P=0.62).

## Conclusions and Discussions

Biobanks have emerged as central tools for biomedical research, yet their true impact remains largely underappreciated and unexplored due to a lack of comprehensive data and metrics (44). While the need for a biobank impact factor has been acknowledged for years (19), our study introduces a comprehensive measure of the multifaceted impact of biobanks. We address long-standing challenges, including the absence of a centralized biobank repository, limited data on biobank impact, and a lack of standardized practices for biobank recognition.

Our analysis reveals that impact is significantly underestimated by traditional metrics, as reflected by the fact that 41.2% of articles fail to cite biobanks’ papers. To measure the true impact, we scanned explicit biobank mentions and measured impact across multiple dimensions—including funding, innovation, and public policy. This comprehensive approach provides empirical evidence to test theoretical insights. Here, we found that the recognition mechanism of biobanks is based on “coauthorship for access,” a result that aligns with previous survey reports (50). More generally, the approach presented here lays the foundations for a more holistic quantification of scientific impact, paving the way for future studies in the science of science. Looking ahead, the integration of language models capable of “understanding” the context of biobank mentions by distinguishing between the explicit usage or informal reference of a resource, ultimately leading to more precise quantification of its contributions to research (57, 58).

## Supplementary Material

Appendix 01 (PDF)

## Data Availability

CSV files have been deposited in Quantifying biobanks and cohort studies (34).
