# Supplementary material for: Quantifying the impact of biobanks and cohort studies
Source: Proc Natl Acad Sci U S A. 2025 Apr 16;122(16):e2427157122. doi: 10.1073/pnas.2427157122 (PMC12037000; doi:10.1073/pnas.2427157122)
Supplement: Supplementary file 1 — Appendix 01 (PDF) [file pnas.2427157122.sapp.pdf]

Quantifying the Impact of Biobanks and Cohort Studies

Supplementary Information

Rodrigo Dorantes-Gilardi et al.

Contents

SI 1 Biobank dataset . . . 4
  SI 1.1 Biobank Catalogs . . . 4
  SI 1.2 Biobank names expansion . . . 6
SI 2 Biobank mentions . . . 7
  SI 2.1 Removing Biobank Duplicates . . . 7
  SI 2.2 Scientific articles . . . 8
  SI 2.3 Patents . . . 9
  SI 2.4 Grants . . . 9
  SI 2.5 Clinical trials . . . 9
  SI 2.6 Public Policy Documents . . . 9
SI 3 Data Validation . . . 9
SI 4 Co-citation network . . . 11
  SI 4.1 Network communities . . . 12
  SI 4.2 Comparison with global MeSH disease distribution . . . 14
SI 5 Hidden Citations . . . 16
  SI 5.1 Reference papers . . . 16
  SI 5.2 Biobank Institution and Team . . . 16
  SI 5.3 Biobank mentions . . . 17
SI 6 Null model . . . 18
SI 7 Disease Impact . . . 18
  SI 7.1 MeSH diseases . . . 20
  SI 7.2 UK Clinical Research Collaboration: UKCRC Diseases . . . 22
  SI 7.3 Research, Condition, and Disease Categorization (RCDC) . . . 22
SI 8 Mentions data . . . 23
  SI 8.1 Publication data . . . 23
  SI 8.2 Grants data . . . 27
  SI 8.3 Patents data . . . 27
  SI 8.4 Clinical trials data . . . 28
  SI 8.5 Public policies data . . . 28
SI 9 Biobank data . . . 28
  SI 9.1 Genetic data . . . 29
  SI 9.2 Environment data . . . 29
  SI 9.3 Follow-up data . . . 29

SI 9.4 Medical records 29
SI 9.5 Registries and disease-specific data 30
SI 9.6 Surveys and Questionnaires 30
SI 9.7 Age groups 30
SI 9.8 Country of origin 30
SI 9.9 Open Data Index 30
SI 9.10 Cohort size 31
SI 9.11 Population vs. health based biobanks 32
SI 10 Biobank Impact Factor 32
SI 10.1 Testing different weights to compute the bIF 33
SI 11 Generalized Linear Model 35
SI 12 Code Availability 37

SI 1 Biobank dataset

Biobanks and cohort studies are closely intertwined, often leading to interchangeable use. Typically, a cohort study is initiated along an associated biobank, maintaining the study's original name. Conversely, some biobanks are designed to support multiple cohort studies, showcasing their versatile application in research. Here, we use the term biobank independently from the process and history responsible for its creation. Estimating the total number of biobanks worldwide is challenging due to the lack of a comprehensive, centralized data source. A recent study estimated around two biobanks per one million people globally, suggesting approximately 660 biobanks in the USA alone [1]. Other estimates place the global number in the realm of just a few hundred biobanks [2]. The existing biobank directories are often limited in scope, covering specific regions or research projects with varying methodologies [3]. Further complicating biobank tracking is the issue of name multiplicity, where typing errors or references to secondary collections lead to inconsistent naming conventions [4] as well as inconsistencies on what constitutes a biobank [5]. Despite these challenges, attempts to quantify and catalog biobanks remain crucial for assessing their overall impact on research and healthcare.

The process to build a corpus of biobank names was based on two steps. First, we compiled a set of 16 biobank catalogs (SI 1.1), and second, we extracted biobank names from the text of 3,310,320 biobank-related articles (SI 1.2). After each step, we processed the names identified to find and remove duplicates based on word similarity and co-appearance in articles of each pair of names (SI 2.1). The resulting corpus of biobanks contains 2,663 unique biobank names that were then used to gather biobank mentions from textual documents (SI 8). Finally, the dataset was validated using an external database of GWAS studies (SI 3).

The full dataset, as well as the code to collect and analyze it, are available at `github.com/Barabasi-Lab/quantifying_biobanks` and `10.5281/zenodo.11671294`.

SI 1.1 Biobank Catalogs

Although there is yet no central biobank repository including all biobanks, there have been several efforts to build catalogs for the scientific community. The largest to date is the BBMRI-ERIC Directory of Biobanks and their collections. The resource was established to "improve accessibility and interoperability between academic and industrial parties to benefit personalized medicine" [6]. As of 10/2023, the directory included a list of 617 European biobanks together with their 3,176 collections and related metadata, providing a large set of biobank names.

To complement this corpus, we integrated the biobank names found in 15 prominent directories of population bioresources (the resulting corpus of biobanks can be found

Table SI 1: Collected Features of Biobanks

Type	Features
Cohort	Size, age group, origin, type (population/health-based)
Data	Genetic (Whole-genome sequencing, GWAS, or environmental), socioeconomic, follow-up, medical records, surveys, registries
Papers	MeSH identifiers, RCDC categories, HRCS classification
Patents	Cooperative Patent Classification codes, assignee
Grants	NIH activity codes, USD amount, funders
Clinical Trials	Study type, RCDC categories
Public Policy	Organization type, country

in raw and processed formats in the data folders 'cohorts' and 'raw_cohorts', respectively). Specifically, this dataset includes biobanks from BIOLINCC [7], EPND [8], IADRP [9], Molgenis [10], P^3G (Public Population Project in Genomics and Society) [11], DCEG [12], DPUK [13], UKRI cohort directory, Birthcohorts [14], CEDCD [15], The Pooling Project of Prospective Studies of Diet and Cancer [16], Wikipedia's list of biobanks, SciCrunch [17], Maelstrom [18], dbGAP [19], and JPND [20]. The resulting list contained 1,576 unique names. Most of these resources were available for download with the exception of Birthcohorts, IADRP, Pooling Project of Prospective Studies of Diet and Cancer, and UKRI, for which we crawled the websites (code found at `python/crawl`).

Furthermore, we extracted the biobank names found in the titles of papers published in the 'Cohort Profile series' of the International Journal of Epidemiology and BMJ Open Journal, both having strict guidelines to start the title with 'Cohort Profile:', followed by the biobank name. We identified these publications using regular expressions on the full publication dataset of Dimensions (files `SQL/cohort_profile.sql` and `SQL/cohort_profile2.sql`), resulting in 488 publications containing 368 biobank names.

Finally, we used ChatGPT to generate a list of biobanks using the prompt 'produce a list of biobank names along with their country of origin', resulting in 738 names. After processing to remove special characters, trailing spaces, and parenthesis, the resulting list contained 2,207 unique biobank names (file `data/cohorts/`

final_cohorts.csv). The code to produce the list is found at python/cohort_names/raw_cohorts.py.

SI 1.2 Biobank names expansion

In order to expand our biobank list, we did a bibliography search based on a set of 3,310,320 biobank-related publications. These publications were obtained by first identifying 162,988 articles mentioning one of the 2,207 biobanks previously obtained and then extracting their citing articles. The resulting set of publications then either mentions a biobank directly or cites a paper mentioning one. The corpus of mentioning publications was obtained by searching in the title, abstract, and acknowledgments sections of articles in Dimensions (SQL/mentions/publications.sql). The code to obtain the set of biobank-related publications is SQL/expansion/papers_citations.sql.

We used regular expressions based on common keywords found in the names of biobanks and similar repositories, including Biobank, Tissue Bank, Registry, Biorepository, Project, and 12 other keywords. The complete list of regular expressions, as well as the code to obtain the potential biobank names, can be found at SQL/expansion/clean_expanded_cohorts.sql. The resulting list of names yielded 23,435 potential biobank names. We filtered potential biobanks in this corpus of names based on each biobank's number of mentions in two different corpora: the set of biobank-related papers (N=3,310,320) and the set of papers mentioning a biobank from the catalogs (N=162,988). A potential biobank was removed from consideration if less than 5% of the articles mentioning it contained a mention of another biobank from the catalogs and less than 50% of its mentions came from biobank-related publications. The code to obtain papers mentioning the potential biobanks is in SQL/expansion/expanded_papers.sql (set of papers mentioning a potential biobank) and SQL/expansion/expanded_papers_internal.sql (set of mentions from biobank-related papers). The code to obtain their number of co-mentions with biobanks from the catalogs is at SQL/expansion/clean_expanded_cohorts.sql, and the code to calculate the percentages of mentions on each corpus is SQL/expansion/cohorts_counts.sql.

This expansion process resulted in an additional 1,924 biobank names (file data/expansion/selected_cohorts.csv), resulting in a total of 4,131 biobanks. The code to clean, preprocess, and obtain this list of biobanks is found at python/expansions/total_inner_ratio.py.

SI 2 Biobank mentions

In order to assess the impact of biobanks we searched their mentions across multiple textual documents, from the Dimensions academic database we include scientific publications, clinical trials, grants, and public policy documents. From Google Patents Public Data, we searched for patents mentioning biobanks, as they provide the full text of each patent. The search for mentions was case insensitive and included the name of the biobank preceded by the word 'the' to reduce false positives. The code to search for mentions can be found in multiple files named after the type of document they were searched on in the folder `SQL/mentions/` for 2,207 catalog biobanks, and `SQL/expansion/mentions/` for the expanded list of 1,924 biobanks.

To focus on biobanks with traceable presence in biomedical science, we remove those without at least one mention in either the title, abstract, or acknowledgments section of a scientific publication. This included 1,163 biobanks from the catalogs and 1,882 biobanks from the expanded list, for a total of 3,045 biobanks.

SI 2.1 Removing Biobank Duplicates

A common issue with biobanks is the multiplicity of names a single biobank can accumulate across publications. The reasons behind this issue are multiple: from name alterations in follow-up studies (e.g., Framingham Heart Study, Framingham Children's Study, and Framingham Offspring Study), use of abbreviations (e.g., ARIC instead of Atherosclerosis Risk in Communities), to inconsistencies in their naming across publications (e.g., Northern Sweden Medical Biobank and Medical Biobank of Northern Sweden or Leeds Biobank and Leeds Multidisciplinary Research Tissue Bank). Additionally, biobanks and their corresponding cohort studies can be used interchangeably, like in Lifelines Biobank and Lifelines Cohort Study, similarly to biobanks that are part of a consortium (e.g., African Neurobiobank for Precision Stroke Medicine and H3Africa).

In order to minimize the number of name pairs referring to the same biobank, we performed a deduplication process based on two factors: (i) string similarity and (ii) co-mentions. The former allows the identification of similar names of a single biobank and the latter to identify duplicates lacking string similarity, such as abbreviations from their full name. String similarity of each biobank pair a and b was calculated using the Indel distance $I(a,b)$, measuring the minimal number of insertions and deletions needed to transform one string into the other, normalized by the maximum number of possible deletions and insertions ($I(a,b) = 1$ implying that $a = b$). We used two variants of these distances: the partial ratio I_{pr}, measuring the Indel distance needed to match the smallest string into a sub-sequence of the larger string (here $I_{pr}(a,b) = 1$ does not necessarily imply that $a = b$); and the token set ratio I_{sr}, based

on the minimal number of word deletions and insertions needed to match a and b (the word order is irrelevant). Using Spacy, a natural language model, we 'cleaned' the names prior to their analysis to remove coordinating conjunctions, auxiliaries, punctuation, symbols, and numerals.

Next, we measured the co-mention similarity of each biobank pair across the 228,761 publications and 19,299 patents mentioning biobanks. The co-mention similarity $S(a, b)$ between two biobanks a and b is equal to the proportion of documents mentioning a that also mention b in their text. In other words, $S(a, b) = 1$ implies that a is exclusively mentioned in publications mentioning b, and $S(a, b) = 0$ that no publication co-mentions a and b. Note that S is not symmetric: for cases where mention counts of biobanks a and b differ, we have that $S(a, b) \neq S(b, a)$.

Finally, we identified a pair of biobanks a and b as duplicates if at least one of the following statements occurred: (i) $\min\{S(a, b), S(b, a)\} > 0.2$ and $I_{pr}(a, b) > 0.9$, (ii) $\min\{S(a, b), S(b, a)\} > 0.05$ and $I_{pr}(a, b) = 1$, (iii) $\max\{S(a, b), S(b, a)\} > 0.3$ and $I_{pr}(a, b) > 0.9$, (iv) $\max\{S(a, b), S(b, a)\} > 0.1$ and $I_{sr}(a, b) = 1$, where co-mentions where extracted from publications. For patent-based similarity, we only considered a and b to be duplicated if all three inequalities were true: $\max\{S(a, b), S(b, a)\} > 0.4$, $\min\{S(a, b), S(b, a)\} > 0.3$, and $I_{pr}(a, b) = 100$, where stricter similarity conditions accounted for a higher probability of co-mentions in full-text documents.

The deduplication process resulted in 337 duplicate pairs, often forming duplicated groups where a biobank had 3 or more alternative names (An example is the Framingham Heart Study or FHS, with 10 variations). To obtain all duplicate groups, we constructed the duplicate network in which nodes represent biobank names and edges duplicated pairs, and each connected component represents name variations of the same biobank. We completed the deduplication process by selecting the biobank name with the greatest number of article mentions in each duplicate group to be the name variation to replace the others.

After deduplication, the final biobank corpus contained 2,263 names mentioned across 228,761 articles, 16,210 grants, 15,469 patents, 1,769 clinical trials, and 9,468 public policy documents. The code containing the deduplication process is found at `python/cohort_names/deduplicate.py`.

SI 2.2 Scientific articles

In order to look for mentions across Dimensions' publications (from October 2023), we first processed the text from the title, abstract, and acknowledgments sections to remove Unicode characters, double spaces, commas, and periods. The code is found at `SQL/clean_text/publications.sql`. Once the articles' text is pre-processed, we look for textual mentions of biobanks within the title, abstract, and acknowledgment sections of 141,219,539 articles, resulting in 250,857 biobank-article mentions

on 228,761 articles (`data/expansion/cohort_patents.csv`).

SI 2.3 Patents

We use the publicly available Google Patents Open Data to search for biobank mentions on the full text of 153,696,878 patents. The search was done using Google BigQuery on the table `patents-public-data.patents.publications`, resulting in 18,183 biobank-patent mentions on 15,469 unique patents (`data/expansion/cohort_patents.csv`. The patents found were then merged to the patent data from Dimensions using the grant application number id (`SQL/join_dim_google_patents.sql`).

SI 2.4 Grants

We used grant data from Dimensions, data composed of 5,040,039 grants. We found 18,251 biobank-grant mentions on 6,210 unique grants (`data/expansion/cohort_grants.csv`).

SI 2.5 Clinical trials

We used clinical trials from Dimensions, data composed of 801,708 clinical trials. We retrieved 1,881 biobank-clinical-trial mentions from 1,769 unique clinical trials (`data/expansions/cohort_clinical_trials.csv`).

SI 2.6 Public Policy Documents

We used the public policy documents from Dimensions, a dataset composed of 1,783,533 public policy documents. We identified 10,285 biobank-public-policy mentions on 9,468 unique public policy documents (`data/expansions/cohort_public_policy.csv`).

SI 3 Data Validation

To assess the comprehensiveness of our database of biobanks and related documents, we used the NHGRI-EBI Catalog of Genome-Wide Association Studies (GWAS) as a reference to measure the scope of our data (the catalog was accessed June 2024), compiling more than 100,000 genetic associations to disease from 6,899 research publications [21]. Indeed, biobanks and population studies play a major role in the discovery of biomarkers by providing samples and genetic data used in GWAS. Yet, they are not the only contributors: cross-sectional and case-control studies—largely

found in clinical trials and not really considered in our dataset—remain the most used resources for biomarker discovery [22]. Another potential caveat to consider is our lack of full-text publications for our search, a limitation not present in the reference GWAS catalog, directly receiving its data from the authors of the articles.

To be able to compare the overlap between the two datasets, we used Dimensions to search the PubMed IDs of our 228,761 biobank-related publications, successfully doing so for 76.6%, but dropping 1/4 of the biobank-related papers in the process. We start the overlap assessment with a naive analysis by directly considering all 6,899 publications from the NHGRI-EBI catalog, finding that 2,602 GWAS publications, or 38% of the total, are also included in our publication data. In our dataset, these publications collectively mention 486 biobanks, the most mentioned by far being the UK Biobank with 748 publications, followed by FHS (169), Wellcome Trust Case Control Consortium (166), and the Rotterdam Study (156).

To measure our dataset's breadth in the reported mentions by individual biobanks, we compared them to the reported numbers found in the NHGRI-EBI Catalog. Specifically, we extracted the number of publications that each biobank in the catalog is referenced as a data source and compared it to the number of mentions we found for that biobank across the catalog's publications. We calculate these numbers for 89 biobanks having the same name in both datasets, finding that our dataset identifies, on average, 10.3 more publication-biobank associations than NHGRI-EBI. The UK Biobank is found in 748 publications in our dataset but only in 485 publications of the reference catalog, a difference showing a much higher breadth in our dataset for this Biobank. The 263 publications mentioning the UK Biobank that do not list this biobank in the reference catalog have 'NR' (230) missing values for the column 'cohort' (33), suggesting that authors of the GWAS studies did not include the biobank source in the NHGRI-EBI Catalog. On average, the 72 biobanks better or equally covered in our dataset have 14.4 missing publications in the reference catalog. On the other hand, the 17 biobanks that are better covered in the NHGRI-EBI Catalog have 7.1 missing publications in our dataset.

Removing publications without a biobank assigned (cohort values 'NR', 'other', or 'NA'), our dataset covers 60% of the 727 remaining publications in the GWAS catalog. These results show a good coverage of our dataset, not without its limitations: while not all of the GWAS publications not covered (40%) may have a population biobank as a data source, we miss mentions in the full text of the paper. To have an approximate percentage of missing mentions due to our lack of access to the full text of a publication, we consider the 485 publications in the GWAS catalog identifying the UK biobank as their source. Of these, 129 are not covered in our dataset, suggesting that 26% of mentions of the UK Biobank may only be found in the body of the manuscript (excluding title, abstract, and acknowledgments). The data, including the number of covered and missing publications, is `data/database/meta/coverage.csv`

and the code for the coverage analysis at `python/cohort_metadata/gwas_papers.py`.

SI 4 Co-citation network

The biobank co-citation network is constructed using the publications mentioning biobanks, where the set of publications of a biobank is composed of those mentioning its name in their title, abstract, or acknowledgments sections. Co-citation is a measure of similarity between two publications. We chose this measure for its flexibility to capture similarity patterns based on direct citations as well as on the citing papers themselves [23]. The use of co-citation also addresses the problem of having to measure the similarity between two biobanks with a few mentions each, as the edge weight (level of similarity) is given by the set of articles citing them instead of the papers mentioning them.

The nodes of the network correspond to biobanks, and two biobanks are connected by an edge if their corresponding publications are often cited together. We operationalize the citations of a biobank as the articles citing at least one of the publications mentioning the biobank. Mathematically, let $C_i = i_1, i_2, \ldots i_n$ be the citing articles of biobank i, and $C_j = j_1, j_2, \ldots j_m$ the citing articles of biobank j, then the union $C_i \cap C_j$ is composed by the articles citing both biobanks i and j. For each biobank i, we only consider its most similar biobank by adding a single edge to the biobank j such that $|C_i \cap C_j| = \max_x |C_i \cap C_x|$, for each biobank $x \neq i$. In other words, each biobank i is connected by an edge to the biobank j that is more often cited together with i. We assign a weight to each edge equivalent to the number of common citations between the two biobanks.

We chose to connect each biobank only to its most frequently co-cited partner to avoid potential biases from highly-mentioned biobanks, where even random co-citations between popular biobanks could obscure meaningful similarities between less frequently mentioned ones. For example, the UK Biobank (UKB) and the Nurses' Health Study (NHS) share 9,795 co-citations despite having different research focuses (NHS primarily studies breast cancer while UKB focuses on genetic chronic diseases). In contrast, NHS and the Black Women's Health Study (BWHS), which both focus on breast cancer research in different populations, share 2,689 co-citations. Even when normalizing edge weights by the total mentions of each biobank ($|C_i \cap C_j|/(|M_i|+|M_j|)$, where M_i is the set of articles mentioning biobank i), the UKB-NHS connection (0.71) would appear stronger than the more scientifically aligned NHS-BWHS connection (0.69). By retaining only the strongest connection for each node, we better preserve scientifically meaningful associations - for instance, NHS connects to the Health Professionals Follow-Up Study (HPFS), an all-male study de-

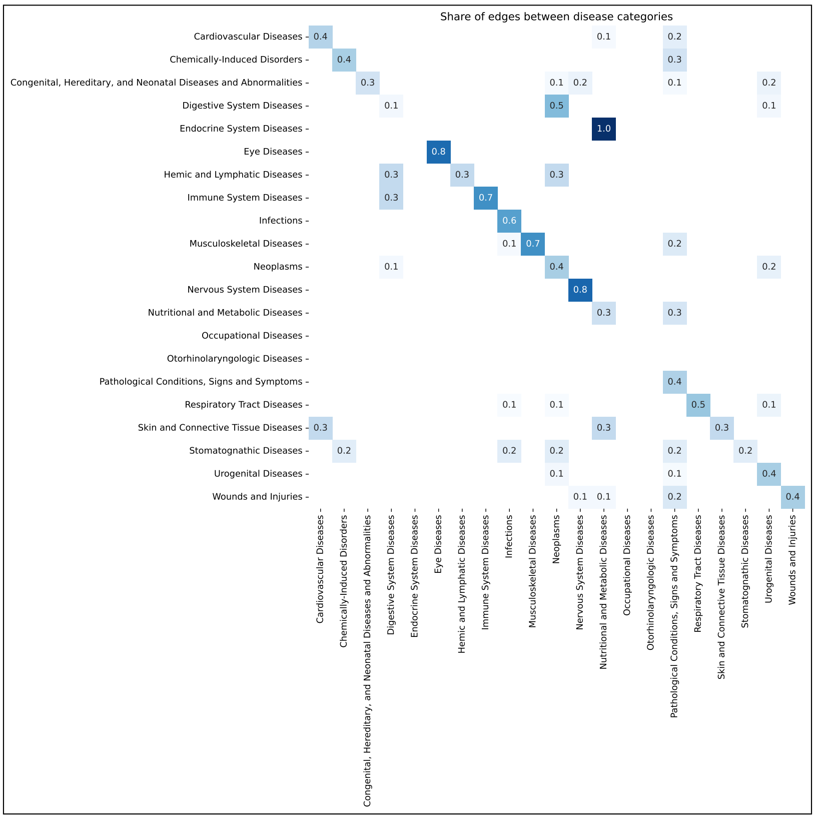

Figure SI 1: Share of edges between MeSH categories in the co-citation network. Each row represents the proportion of edges that connect with each disease category relative to the number of edges linking to a node in that row's category. Only cells with at least 10% of the edges of that row's category are shown.

signed to complement the all-female NHS; UKB links to the Wellcome Trust Case Control Consortium (WTCCC), another UK-based resource for studying genetic architecture of diseases; and BWHS maintains its connection with NHS.

SI 4.1 Network communities

In assess the modularity the biobank co-citation network, we use a community detection algorithm to uncover 30 communities using the Louvain method [24], a modularity-maximization method with the offset of merging small sub-networks when the resolution parameter is low [25]. The corresponding node partition has a Girvan-Newman modularity score of 0.879, suggesting dense connectivity within the communities [26]. To compare the overlap between the communities and the 20 annotated categories based on the main MeSH disease of each node, we first construct a contingency table (Figure SI 3) where each row represents a disease category and each column represents

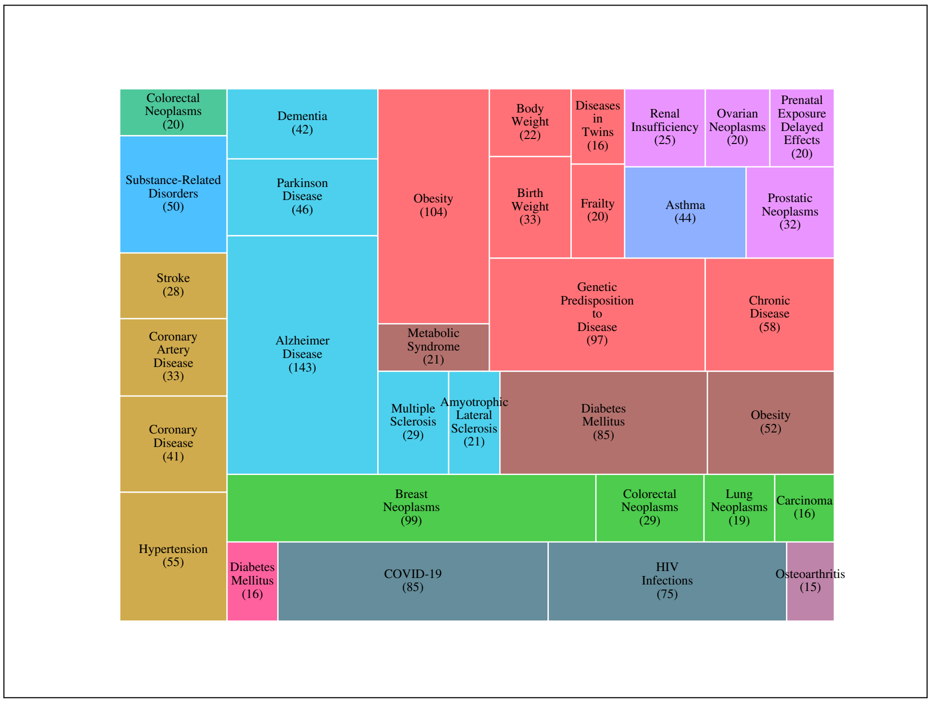

Figure SI 2: The disease focus of biobanks. Based on the fifth-level disease categories of the MeSH tree (e.g. Alzheimer Disease, with tree identifier "C10.574.945.249" and separated by 5 steps from the tree root), we identify the disease focus of each biobank by extracting the most studied condition in publications that mention the biobank. The total area of the rectangle represents all biobanks, and each sub-rectangle's area is proportional to the share of biobanks (absolute number shown in parenthesis) focusing on the corresponding disease.

a community detected by the algorithm [27].

Based on this table, we then calculated three metrics: the normalized mutual information (NMI) score [28], capturing the mutual dependence between the two classifications [29]; the overlap percentage O_p, a measure of how many nodes share the same classification in both classifications; and the Chi-square test score χ^2 and p-value, determining whether there a statistically significant relationship between the community structure and the disease categories. We find that the relationship between communities and disease categories is highly statistically significant ($\chi^2 = 5235$, p-value $< 10^{-3}$). However, we find a modest normalized mutual information score (NMI = 0.247), suggesting that while there is some meaningful relationship between the community structure and disease categories, the community structure is not captured by disease categories alone. This is confirmed by the overlap percentage ($O_p = 43.4\%$), indicating that when we look at each detected community and identify its most common disease category, four out of ten biobanks share their community's predominant disease focus.

SI 4.2 Comparison with global MeSH disease distribution

To assess whether the observed concentration of biobank research in certain disease categories reflects broader patterns in biomedical research, we compared the distribution of MeSH disease categories in 111,525 biobank-related publications (SI 7.1) with the overall distribution across 31,170,650 articles with MeSH headings in the biomedical literature published before 2023. Our analysis revealed strong correlations between the number of papers per disease category in biobank research and general biomedical research (Pearson correlation $r = 0.76$, p-value $= 4 \times 10^{-5}$; Spearman correlation $\rho = 0.88$, p-value $= 6 \times 10^{-8}$). These high correlations suggest that the focus of biobank research on certain diseases largely mirrors the general trends in biomedicine. However, we identified several notable differences in the relative representation of certain disease categories. Some categories were significantly underrepresented in biobank research compared to the general biomedical literature, namely Infections (4.4% of biobank articles vs 12.5% of total articles) and Cancer (12.7% of biobank articles vs 20.8% of total articles). Conversely, other categories showed substantial overrepresentation in biobank research: Cardiovascular diseases (15.0% of biobank articles vs 8.7% of total articles) and Nutritional diseases (14.3% of biobank articles vs 4.11% of total articles).

These differences highlight how biobank research, while generally following broader biomedical research patterns, has developed distinct areas of focus. The overrepresentation of cardiovascular and nutritional diseases may reflect the historical strengths of population-based cohort studies in these areas, while the underrepresentation of infections and cancer could indicate opportunities for expanded biobank research in

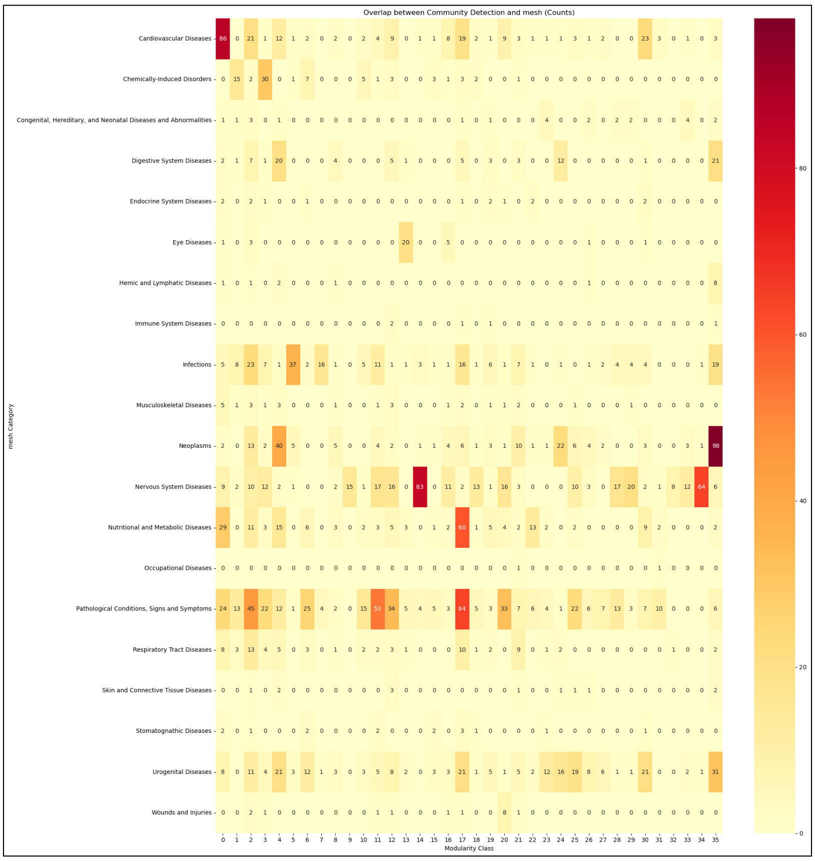

Figure SI 3: Contingency table comparing communities with disease classification.

these fields.

SI 5 Hidden Citations

Hidden citations are a measure of the hidden impact of a biobank based on its reference papers [30] and refer to unambiguous allusions made to a biobank without a corresponding citation. The idea is rooted in eponyms [31], informal citations, and "obliteration by incorporation" [32]. In the context of biobanks, these allusions appear as implicit citations based on contextual mentions of a biobank in the text of a scientific paper. A hidden citation to a biobank is the difference between the explicit citations from papers mentioning the biobank and its total number of mentions.

SI 5.1 Reference papers

In order to compute hidden citations, we first identify the foundational or reference papers of each biobank from the pool of papers that mention it [30]. We identify a paper x a reference paper of a biobank if x mentions the biobank in its title or abstract and either (1) x is the first publication mentioning the biobank; (2) a paper y references x with probability $P(x|y = m) > 0.2 + P(x)$, where $P(x|y = m)$ is the probability that y references x given that y mentions the biobank and $P(x)$ is the probability that x is cited by any paper; or (3) the title of x contains the words 'design', 'baseline characteristics', or 'rationale'. We identify at least one reference paper for 500 biobanks. The code to compute the missing citations is found at `python/impact/recognition/foundational.py`. The data can be found at `data/expansion/database/cohort_impact/reference_papers.csv`.

SI 5.2 Biobank Institution and Team

To identify the institution associated with each biobank, we analyze the affiliation patterns of the principal investigators (PIs) across all reference papers. For each biobank, we first identify its core research team by examining the authorship of its reference papers (described in SI 5.1). From this set of authors, we identify PIs as researchers who either: (1) appear as corresponding authors in papers where a significant proportion ($> 10\%$) of citations are to other papers mentioning the same biobank; or (2) contribute to a substantial portion ($> 5\%$) of all papers mentioning the biobank. For each biobank, we extract the organizational affiliations of these PIs from author metadata available in our database.

The primary institution of a biobank is determined by identifying the most frequently occurring organizational affiliation among its PIs. In cases where multiple

affiliations appear with equal frequency, we select the institution associated with the PI whose papers have received the highest citation count. This approach allows us to identify a single primary institutional home for each biobank, even when research teams span multiple organizations. Additionally, we extract the country of each biobank using the same methodology. The institutional and country affiliations enable us to measure the extent to which biobank research impact remains concentrated within the founding organization and country.

The complete biobank team is composed of the union of all authors listed in any of the biobank's reference papers. We further analyze team structure by examining authorship patterns across all papers mentioning the biobank, allowing us to distinguish between core team members who appear consistently across multiple publications and peripheral contributors. This data is stored in structured format and can be found at `data/expansion/database/descriptive/biobank_PIs.csv` for principal investigators and `data/expansion/database/descriptive/biobank_team_20members.csv` for team membership.

SI 5.3 Biobank mentions

Authors may mention a biobank not because they directly used its data, but as part of citing prior research. For instance, consider the hypothetical reference: "Using data from Biobank X, one study demonstrated that drug Y significantly improves outcomes for disease Z [citation]". In such cases, the authors are not required to cite the primary paper describing Biobank X but instead cite the study that used its data. Moreover, this type of reference does not indicate that the author is a user of Biobank X who neglected to cite its official reference(s). This is a limitation in our study, as we do not perform detailed natural language processing to distinguish the type of mention. However, our methodology specifically focuses on biobank mentions in titles, abstracts, and acknowledgments sections ($N = 228,761$ papers) rather than full text, which increases the likelihood of capturing actual biobank usage rather than indirect references (perhaps with the exception of the Methods Section).

To assess the extent of this limitation, we randomly selected 100 instances of biobank mentions along with their surrounding text from 50 abstracts and 50 acknowledgment sections. Each mention was manually classified as either direct (indicating the biobank was a direct data source) or indirect (where the biobank was referenced without directly contributing data). Additionally, we categorized the context in which the biobank was mentioned, such as acknowledgments of participants or explicit references to data usage. Our classification revealed that 93 out of 100 mentions were direct references to a biobank, while only 7 were indirect, with 5 of those attributing the biobank as a funding source rather than a data provider (Table SI 2). Among the direct mentions, 74 out of 93 papers explicitly acknowledged

using the biobank's data (62) or expressed gratitude to its participants (12).

Reasoning	Count	Mention Type
Explicit Usage	62	Direct
Participants thanked	12	Direct
Biobank funded the study	5	Indirect
Paper introducing biobank	6	Direct
Biobank's funding	3	Direct
Biobank acknowledged	4	Direct
Funder thanked	2	Direct
Biobank used as facility and not as data source.	1	Indirect
Review of study using biobank	1	Indirect
Biobank name is parent collection	2	Direct
Biobank name explicitly thanked	1	Direct
Biobank team thanked	1	Direct

Table SI 2: Types of biobank mentions in 100 randomly sampled papers.

SI 6 Null model

In order to test the significance of the results showing that the impact of biobanks is local in terms of affiliation, country, and co-authorship, we create a null model in which we randomize the citing papers of each biobank such that the biobank remains with the same number of citations but with a random set of articles citing it. Once we have a randomized version of the citation network, where citing papers are randomized, we compute the mean share of in-house citations (coming from the same affiliation), the share of same-country citations, and the share of citations containing a collaborator of the biobank's team, and the share of citing papers containing its lead-author. We repeat this process 100 times and then use a z-test comparing the real value of each measure with its respective sequence of 100 randomized values to obtain a p-value.

SI 7 Disease Impact

Disease impact D quantifies a biobank's contribution to medical research based on three key components: disease scope, disease depth, and rare disease impact (Equation 0.1). Disease scope captures how broadly a biobank contributes to research

across multiple conditions, disease depth measures the extent of its impact within major disease groups, and rare disease impact evaluates the proportion of its research dedicated to rare diseases. Each component is derived from biobank-related publications indexed in MeSH (SI 7.1) and the RCDC classification (SI 7.3).

$$D = D_{\text{scope}} + D_{\text{depth}} + D_{\text{rare}} \tag{0.1}$$

Disease scope measures the extent to which a biobank contributes to research across different medical conditions. A biobank that supports studies on a wide range of diseases will have a higher scope score. For each condition c, we compute the fraction of total research publications that involve the biobank:

$$b = \sum_{c=1}^{N} \frac{p_c}{P_c} \tag{0.2}$$

where p_c is the number of biobank-related publications on condition c, P_c is the total number of publications on that condition, and $N = 4,982$ is the total number of conditions considered. The overall disease scope is then standardized as:

$$D_{\text{scope}} = \frac{1}{Y} \cdot \frac{b - \mu_b}{\sigma_b} \tag{0.3}$$

where Y represents the number of years since the biobank's inception, and μ_b, σ_b denote the mean and standard deviation of disease breadth across all biobanks (normalized to $[-1, 1]$ based on the diversity of conditions relative to all biobanks). The UK Biobank leads this metric, contributing to studies on 731 different conditions (Figure SI 4A), followed by NHANES (617), WHI, and the Telethon Network of Genetic Biobanks (TNGB, 199).

Disease depth captures the concentration of a biobank's research within major disease groups. Unlike disease scope, which considers the breadth of conditions studied, disease depth measures the relative impact of a biobank's research in predefined disease categories. We focus on 180 major disease groups, defined as third-level MeSH classifications with at least 100 publications per year, encompassing diseases that share common pathophysiological, etiological, or anatomical features (e.g., Gastrointestinal Diseases). The maximum research concentration across these groups is computed as:

$$m = \max_j \left(\frac{p_j}{P_j} \right) \tag{0.4}$$

where p_j represents the number of biobank-related publications on disease group j, and P_j is the total number of publications on that disease group. This fraction is

then standardized and normalized to $[-1, 1]$ through comparison with all biobanks:

$$D_{\text{depth}} = \frac{1}{Y} \cdot \frac{m - \mu_m}{\sigma_m} \tag{0.5}$$

For disease depth, the Avon Longitudinal Study of Parents and Children (ALSPAC) leads in environmental illness research, with 14% of all publications in 2020 (Figure SI 4B). Other high-ranking biobanks include the Human Microbiome Project (HMP) for neoplastic processes (9% of all publications in 2017) and the China National Genebank database (CNGBdb) for agricultural workers' diseases (7% of publications in 2021).

Rare disease impact evaluates the proportion of a biobank's research focused on rare diseases. To quantify this, we compute the fraction of biobank-related publications dedicated to rare diseases:

$$r = \frac{p_{\text{rare}}}{p_{\text{total}}} \tag{0.6}$$

where p_{rare} represents the number of rare disease publications mentioning the biobank, and p_{total} is the total number of publications mentioning the biobank. This fraction is then standardized and normalized to $[-1, 1]$:

$$D_{\text{rare}} = \frac{1}{Y} \cdot \frac{r - \mu_r}{\sigma_r} \tag{0.7}$$

The St. Jude Lifetime Cohort Study (St. Jude LIFE) ranks highest in rare disease impact, with 101 out of 104 publications (97%) focused on rare diseases (Figure SI 4C). Other leading biobanks include the Swiss Childhood Cancer Survivor Study (SCCSS, 96%), the NCI Childhood Cancer Survivor Study (CSS, 94%), and the Ovarian Cancer Association Consortium database (OCAC, 88%).

The code to compute disease impact is found in `python/impact/cohort_impact_factor/disease_impact.py`.

SI 7.1 MeSH diseases

To systematically associate research articles with diseases, we use disease definitions from the Medical Subject Headings (MeSH) classification. A MeSH term is considered a disease if it falls under the 'C' category of the MeSH tree[1]. Using this classification, we link articles to the diseases they investigate based on the MeSH terms assigned to each publication.

For each disease, we calculate the total number of related articles published per year between 2000 and 2013. Across this period, the dataset consists of 10,486,605

[1] `https://meshb-prev.nlm.nih.gov/treeView`

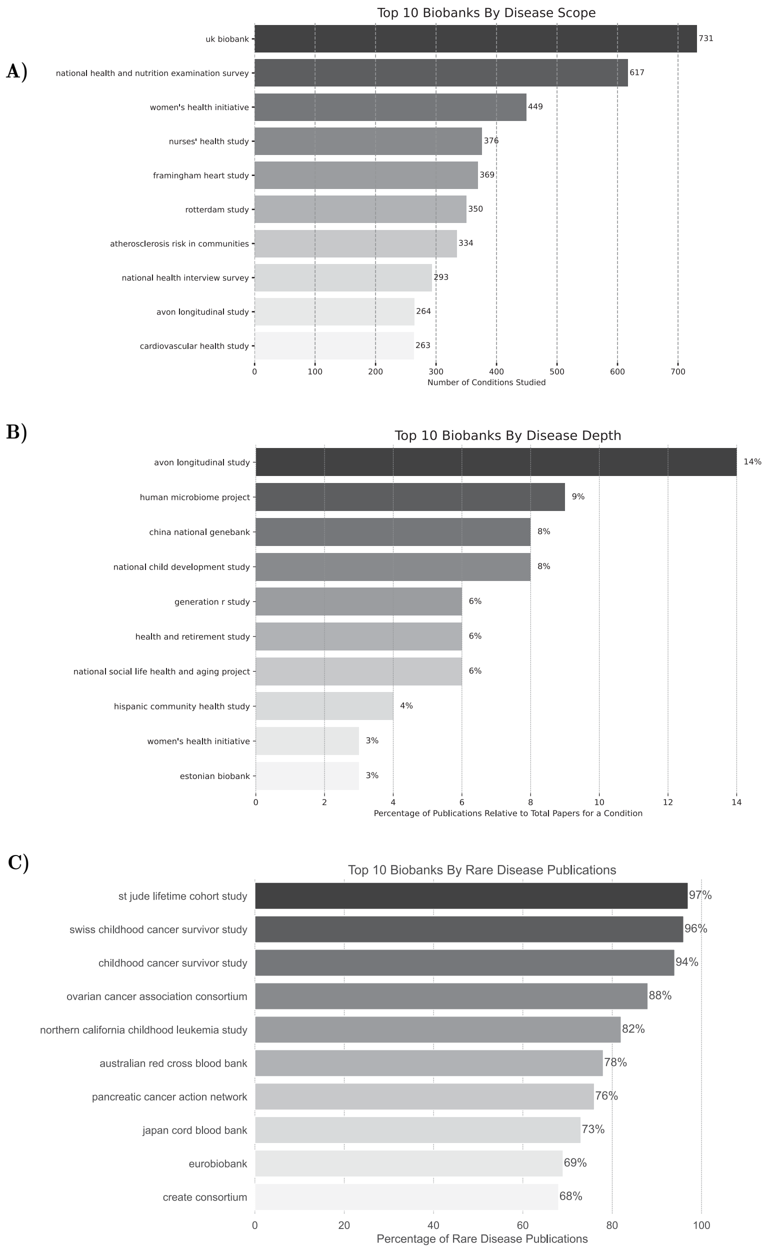

Figure SI 4: The three components of disease impact. Top biobanks by disease impact measure, including A) disease scope, B) disease depth, and C) study of rare diseases.

articles covering 4,982 conditions. To assess the contribution of each biobank to disease research, we compute the proportion of articles mentioning a biobank relative to the total number of publications on that disease in each year when the biobank was active. To ensure statistical reliability, we restrict our analysis to diseases with at least ten publications per year in the global dataset. The resulting dataset captures the proportion of research on each disease in which a biobank is cited.

To compute disease impact, we consider only articles published after 2000 that explicitly mention a biobank, resulting in a dataset of 156,330 papers. Of these, 75,269 papers (48%) are linked to at least one disease, covering 985 biobanks during the study period. The classification of individual diseases and conditions follows the hierarchical structure of the MeSH tree, with broader disease categories encompassing multiple specific conditions. The complete dataset, detailing the main disease categories and subsequent MeSH levels associated with each biobank, is available at `data/expansion/database/cohort_impact/mesh_impact.csv`.

SI 7.2 UK Clinical Research Collaboration: UKCRC Diseases

The HRCS is a classification system used by a large number of health research funders in the United Kingdom (UK) and is subdivided into the Research Activity Classifications (RAC) and Health Categories (HC)[2]. For this study, we considered only the HC categories of the classification, composed of 21 separate categories encompassing all diseases, conditions, and areas of health.

SI 7.3 Research, Condition, and Disease Categorization (RCDC)

This categorization system is used by the NIH to report to Congress and is a biomedical system consisting of 237 categories, some of which are very specific in the topic (e.g., "ataxia-telangiectasia"), and others more general (e.g., "neuroscience"). The advantage of RCDC is the link between each category and its annual funding from the NIH, which allows us to compare research output and funding of each category based on publications mentioning biobanks. For our analysis, we limited our analysis to the 40 categories whose average yearly funding exceeds 1 billion US dollars (USD) and can be linked to at least 100 publications annually. This categorization includes a category for rare diseases, which is used here to identify the publications related to rare diseases mentioning each biobank.

To understand whether commercial interests drive the overrepresentation of certain RCDC categories in biobank research, we analyzed the distribution of categories across 20,088,098 publications, 668,664 clinical trials, and 7,517,131 patents from

[2] `https://hrcsonline.net/health-categories/`

2008-2022. Our analysis revealed distinct patterns between biobank-related documents and the broader biomedical literature.

For patents, aging ranked 6th (1,703 patents) among biobank-related patents across the 40 RCDC categories but only 15th (189,116 patents) in all patents. Similarly, nutrition ranked 13th (964 patents) in biobank-related patents but 6th (472,013 patents) overall. This disparity suggests that commercial interests alone do not explain the overrepresentation of these categories in biobank research.

The distribution in clinical trials shows a stronger alignment with biobank research priorities. Specifically, both aging and nutrition rank significantly higher in biobank-related trials compared to all trials: Nutrition is ranked 3rd (614 trials) in biobank-related trials vs. 14th (54,782 trials) overall, and Aging is ranked 6th (499 trials) in biobank-related trials vs. 16th (56,136 trials) overall.

This pattern suggests that biobank publications in these areas may be primarily driven by medical and clinical applications rather than commercial interests. This interpretation is further supported by correlation analysis between biobank papers and different document types: biobank papers show a stronger correlation with clinical trials (Spearman = 0.9) than with patents (Spearman = 0.71).

Figures SI 5 and SI 6 show the comparative distribution of RCDC categories between biobank-related documents and all documents for patents and clinical trials, respectively.

SI 8 Mentions data

For each document mentioning a biobank, we collect the relevant metadata to implement each of the impact measures developed in the study. For each document type, we leverage different databases that contain further relevant information. This section serves as a summary describing the different databases and the specific information extracted for grants, patents, clinical trials, and public policy documents.

SI 8.1 Publication data

Besides the disease classifications described in a previous section (SI 7), for each publication, we use the Dimensions database to extract its number of citations by year and total, authors, journal venue, references, as well as its citations from other document types, including grant applications, patents, clinical trials, and public policy documents. This allows us to measure not only the impact of biobanks but also the underlying venues and mechanisms propagating their use, e.g., the top journal venues publishing articles mentioning biobanks (Figure SI 7).

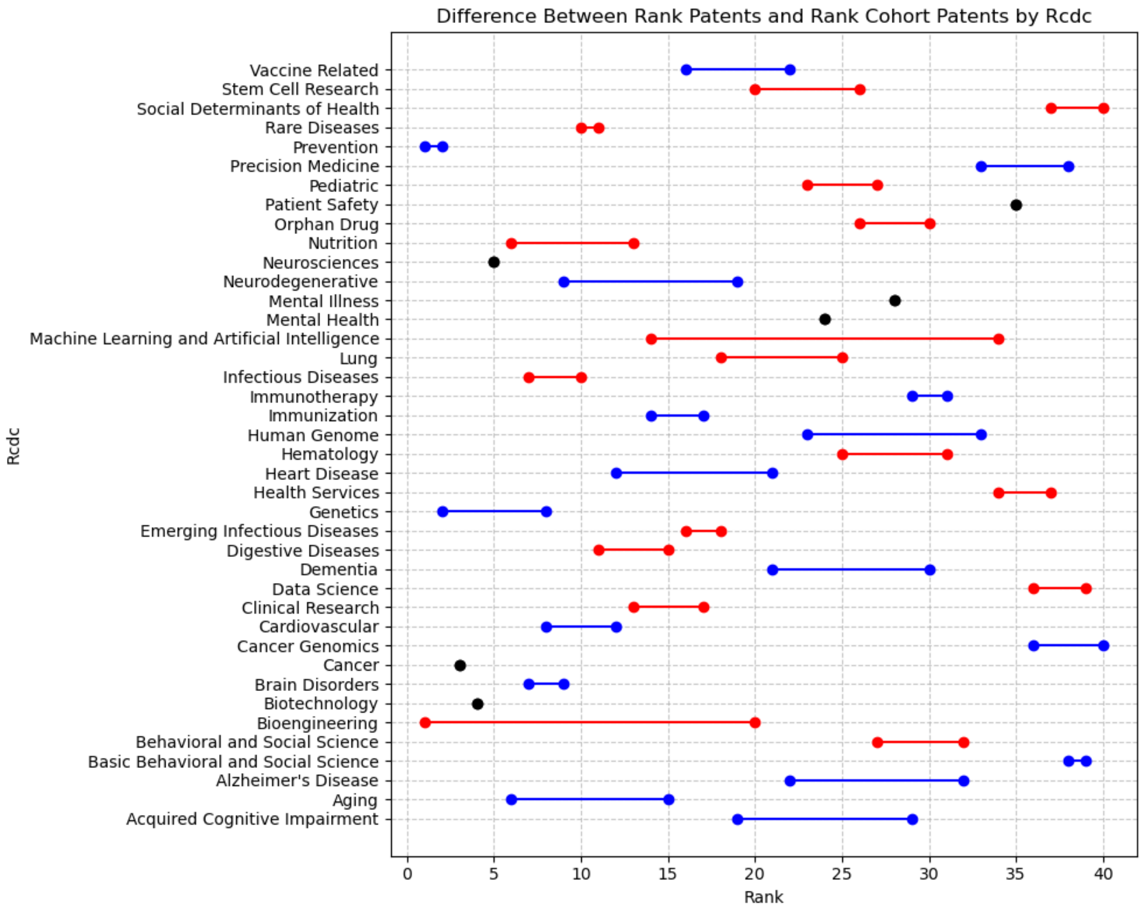

Figure SI 5: Comparison of distribution rank in patents. Global (R_G) and biobank-based ranks (R_B) for 40 RCDC categories based on 7,517,131 general patents and 15,466 patents mentioning biobanks, respectively. An RCDC category with a lower rank has more associated patents than a category with a higher rank. Each line segment's length represents the absolute difference between the two ranks ($|R_G - R_B|$), and are colored red if $R_G < R_B$ (RCDC category is relatively less represented in biobank-based patents compared to general patents) and blue if $R_B < R_G$. Black points represent no change in rank ($R_G = R_B$).

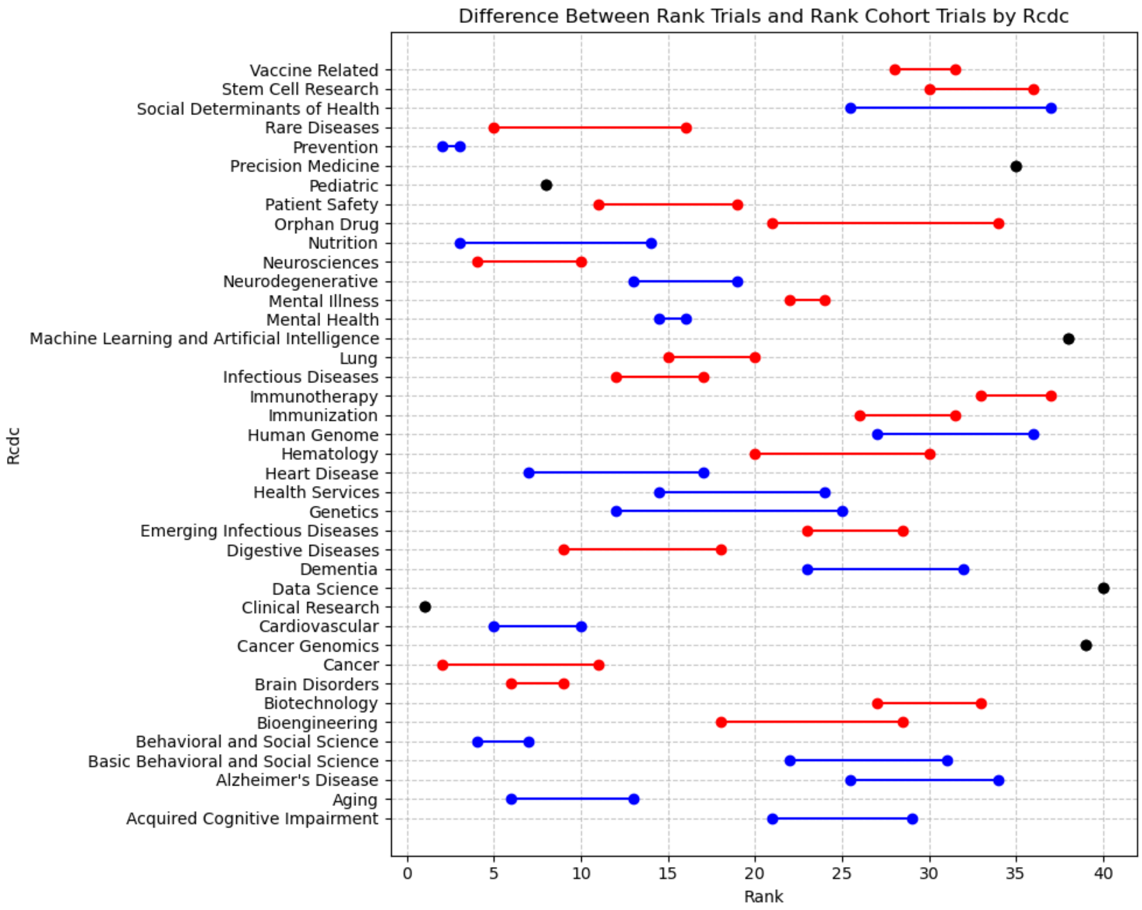

Figure SI 6: Comparison of distribution rank in clinical trials. Global (R_G) and biobank-based ranks (R_B) for 40 RCDC categories based on 668,664 general clinical trials and 1,770 clinical trials mentioning biobanks, respectively. An RCDC category with a lower rank has more associated clinical trials than a category with a higher rank. Each line segment's length represents the absolute difference between the two ranks ($|R_G - R_B|$), and are colored red if $R_G < R_B$ (RCDC category is relatively less represented in biobank-based clinical trials compared to general clinical trials) and blue if $R_B < R_G$. Black points represent no change in rank ($R_G = R_B$).

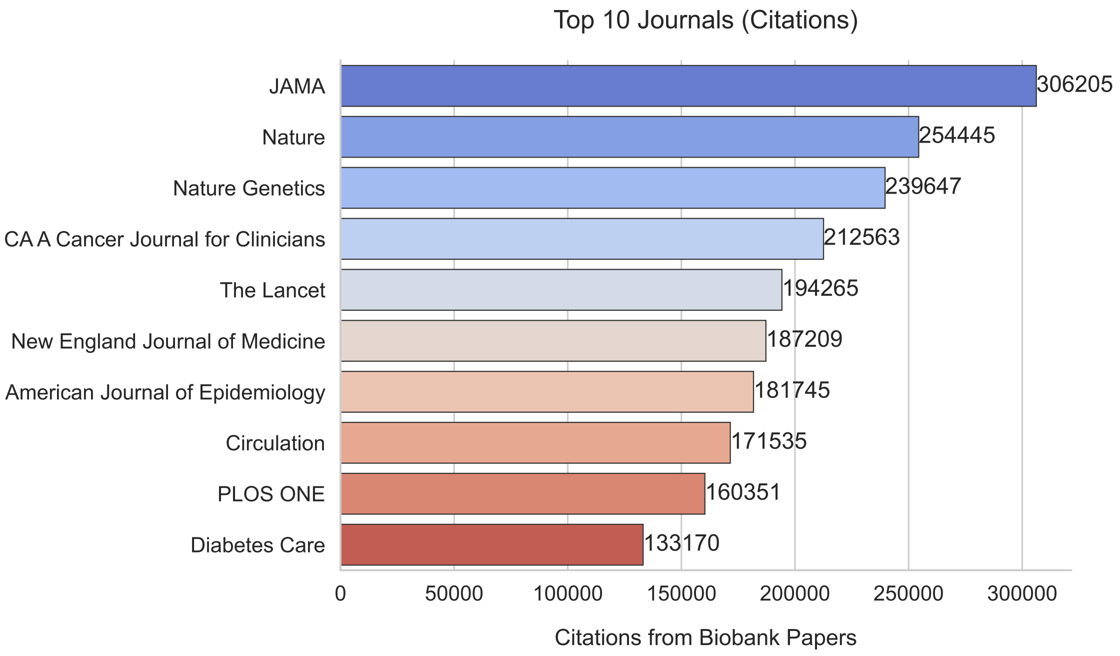

Figure SI 7: Top venues to publish biobank-related research. The summed number of citations of all publications mentioning a biobank yields the citations of each journal.

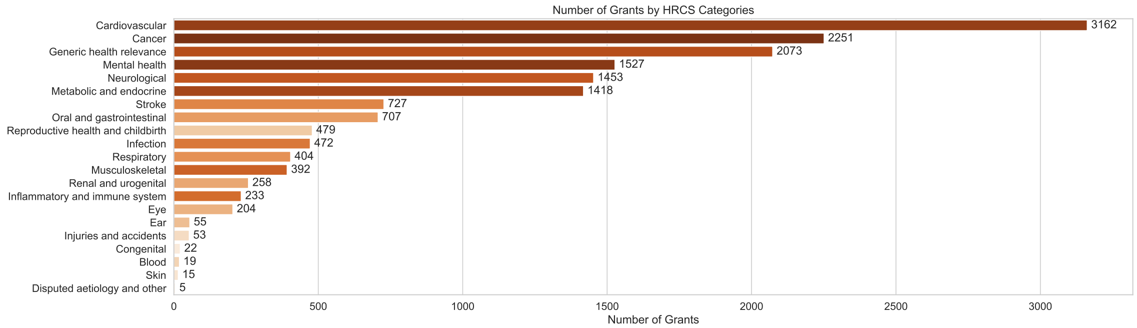

Figure SI 8: Grant distribution by HRCS categories.

SI 8.2 Grants data

For each grant, we extract its NIH activity code, the funder, funder amount, and disease categories (based on MeSH, RCDC, and HRCS, Figure SI 8). The NIH activity codes are then used to identify training, collaboration, and prestigious grants.

Table SI 3: Overview of grants mentioning biobanks

	Total	Biobank Mean (σ)
Grants	16204	14.3 (48.5)
Million USD	30,863	28.3 (115)
Funding Organizations	298	3.4 (5.6)
Funding Countries	37	1.6 (1.5)
Research Organizations	2,421	8.7 (21.3)
Research Countries	74	2 (2.7)
Training grants	1,359	1.3 (6.7)
Collaboration grants	1106	1 (4.6)
Resulting publications	193,884	238.6 (894.4)

SI 8.3 Patents data

We identify each patent's Cooperative Patent Classification (CPC) and its disease categories. Moreover, we extracted each grant's assignee, country, and type. Using the CPC classification, we can study the focus of biobank-related patents, e.g., patents for new drugs (Figure SI 9).

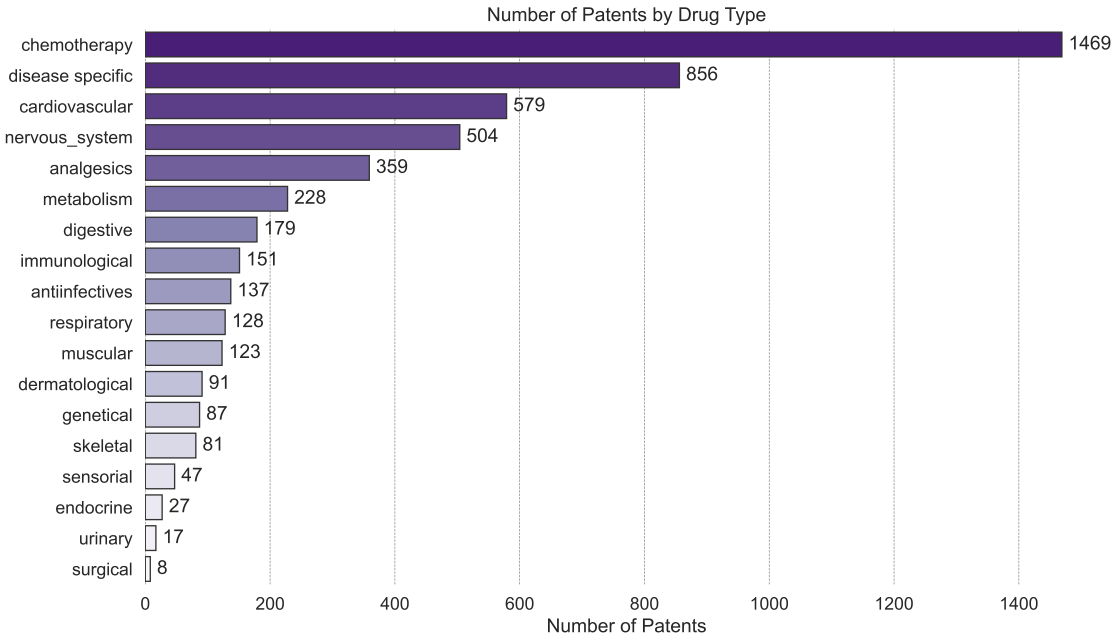

Figure SI 9: Categories of drug-related patents mentioning biobanks.

SI 8.4 Clinical trials data

From each clinical trial, we extract its disease categories, as well as its assignee and its institutional type.

SI 8.5 Public policies data

For each public policy document, we extract its country of publication based on the affiliation of the institution implementing the policy, and the type of its implementing institution.

SI 9 Biobank data

Biobanks differ in the scope and kind of data they offer. A Biobank may focus on a particular physical sample, e.g., brain tissue, while another may offer multiple sample types and questionnaire information from its participants. Genetic biomarkers, those gene variants related to higher risk or diagnosis of a disease, are among the most coveted goals of cohort studies and require DNA data from biobanks. The kind of genetic data, however, can define the methodology used by the study and is therefore

highly relevant. Similarly, medical records can be used to trace risk factors to multiple diseases and conditions suffered by the biobank's participants, complementing its data. Here, we assess the type of data available from the biobank across several factors, including genetic data type, environmental data, follow-up data, medical health records, and disease-specific registry data. In order to identify the type of data available from the biobank, we looked at the MeSH classification terms of the articles mentioning the biobank. If multiple biobank papers are Genome-Wide Association Studies (GWAS), it is highly likely the biobank contains GWAS data available. Here, we present the different MeSH terms we used to associate each data type. The complete set of MeSH terms and tree numbers used can be found at `data/mesh_and_meta/mesh_terms.md`.

SI 9.1 Genetic data

In order to assess whether a biobank has genetic data available, we looked for the following MeSH terms in the articles mentioning it: Genome-wide Association Study, Whole Genome Sequencing, Genetic Association Studies, Genetic Techniques, as well as any MeSH term under the tree classification of Genetic Predisposition to Disease (MeSH tree number C23.550.291.906). We then sub-classified the DNA data using GWAS data and whole genome sequencing. The code can be found at `python/expansion/data/dna.py`.

SI 9.2 Environment data

We used the sub-branches of each of the following MeSH terms to identify papers with environmental data: Gene-environment Interaction, Environmental Exposure, Environmental Biomarkers, Environmental Indicators, Environment, and Environment Design. The code can be found at `python/expansion/data/environment.py`.

SI 9.3 Follow-up data

Often, a biobank's cohort is followed through time, and samples and data are collected on each follow-up. To identify follow-up studies of a biobank, we used the MeSH term 'Follow-Up Studies' (D005500). The code can be found at `python/expansion/data/follow_up.py`.

SI 9.4 Medical records

We used the MeSH terms under the tree classification of 'Medical Records' (MeSH tree ID E05.318.308.940.968). The code can be found at python/expansion `/data/`

medical_records.py.

SI 9.5 Registries and disease-specific data

In order to look for registries and other types of disease-specific data, such as the one coming from panels, we used the following MeSH terms: Registries (E05.318.308.970), National Program of Cancer Registries (I01.409.418.750.600.650.200.760), and Mass Screening (E01.370.500). The code can be found at python/expansion/data/registries_disease_specific.py.

SI 9.6 Surveys and Questionnaires

Some studies rely on data from surveys and questionnaires to build a social profile of the participants of the biobank. To identify those biobanks with survey and questionnaire data we identify the papers under the mesh term Surveys and Questionnaires (E05.318.308.980). The code can be found at surveys_and_questionnaires.py.

SI 9.7 Age groups

Most biobank cohorts are designed with an age group in mind, usually defined by the first wave of data collected from its participants. In order to identify the age group of a biobank, we identified all the age groups under the MeSH classification of Age Groups (M01.060) and classified it into 7 categories depending on the tree number of the age group, namely: Middle Aged (M01.060.116.630), aged (M01.060.116.100), young adult (M01.060.116.815), adolescent (M01.060.057), infant (M01.060.703), child (M01.060.406), and birth (M01.060.261). The code can be found at persons.py.

SI 9.8 Country of origin

We collected the cohort's country of origin from each biobank by identifying the MeSH terms related to countries in their publications. This includes every term under the MeSH tree classification of Geographic Locations (Z01), except for cities (Z01.433). We also consider the countries where the authors of the papers are affiliated based on their institution on each paper. The code can be found at countries.py.

SI 9.9 Open Data Index

To quantitatively assess the level of *data openness* within biobanks, we developed a composite metric that measures how concentrated or distributed research output

is across researchers and institutions. Our Open Data Index considers three key concentration factors derived from the published literature mentioning each biobank: the percentage of papers authored by the single most prolific researcher (P_{author}) measured over their active publication period within the cohort; the percentage of total cohort papers co-authored by the top 10 most prolific researchers (P_{top10}); and the percentage of papers with at least one author affiliated with the most represented research organization (P_{org}).

We then define the Open Data Index (ODI) for each biobank as:

$$\mathrm{ODI} = 100 - \frac{P_{author} + P_{top10} + P_{org}}{3}$$

This formulation ensures that biobanks with more distributed authorship and institutional diversity receive higher ODI scores, reflecting greater data accessibility and collaborative research utilization. Conversely, biobanks where research output is concentrated among a few authors or institutions receive lower scores, suggesting more restricted data utilization. The index ranges from 0 (completely concentrated) to 100 (maximally distributed), providing an intuitive measure of a biobank's openness to broader scientific engagement. The code can be found at `open_data.py`.

SI 9.10 Cohort size

The cohort size of a biobank, i.e., the number of participants or individuals included for sampling, surveys, and/or follow-up, may be of interest to researchers looking to access its data. Although Biobanks usually publish the size of their cohort on their design or foundational papers, it may be subject to change in subsequent updates of the biobank, or it may be a target number. Oftentimes, moreover, researchers may be interested in a sub-sample of the cohort depending on multiple factors such as the disease of interest, the analytical method of their study, or the type of data they need. In order to have an idea of the cohort size that is used across the research papers that make use of a biobank's data, we look for the self-disclosed sample sizes appearing in the abstracts of such studies. This approach makes use of papers mentioning a biobank, resulting in a distribution of sample sizes used across time and topics. In order to identify sample sizes, we use a regular expression matching numbers followed by a keyword (code available at `SQL/expansion/cohort_size.sql`), and to filter out those papers that may be using more than one biobank, we only consider articles mentioning one biobank in our sample. For each biobank, we then consider the 90th percentile as its cohort size to consider the larger sub-samples of the biobank and to avoid outliers that may represent noisy values. The code to create the table with the sample size of each biobank is found at `python/data/cohort_size.py`.

SI 9.11 Population vs. health based biobanks

Biobank data is retrieved from a cohort of participants that may be recruited from a population defined by a geographical location, a line of work, or any other common factor, or it can be based on patients (dead or alive) coming from a hospital or clinic and usually sharing a disease [33]. The border between population and patients, however, is not always clear. This is the case when biobanks recruit individuals from a general population that are patients are local clinics, like in the UK Biobank. Indeed, often, participants are invited to participate in the Biobank's cohort from a clinic or a hospital, not because they are patients there but because it is strategic or convenient (e.g., to link their medical records to their samples).

To assess whether a biobank's design had a population or a group of patients in mind we identified several keywords from the abstracts of the papers mentioning the biobank, including 'population-based', 'patients', 'hospital', 'health-based', and 'clinic'. To classify the cohort type of the biobank, we then computed the ratio of papers with each keyword and assigned the type with the highest proportion of papers. Under this approach, the UK Biobank, even if around 10% of its papers contain the word 'patients', is classified as a population-based biobank as 29% of its articles contain the keyword 'population'. The code can be found at `python/expansion/database/cohort_type.py`.

SI 10 Biobank Impact Factor

We considered two factors to calculate the Biobank Impact Factor (bIF): research mentions and disease impact. To calculate the research impact (R) of a biobank, we standardize its number of mentions for each document type separately (i.e., publications, grants, patents, clinical trials, and public policy documents). This research impact takes into account the overall presence of biobanks across science, innovation, and policy, as well as their lack thereof. For each document type (research publication, grant, patent, clinical trial, and public policy), we calculate a standardized score and then normalize by the biobank's age:

$$R = \frac{1}{Y} \sum_{i=1}^{5} \frac{r_i - \mu_i}{\sigma_i} \tag{0.8}$$

where r_i is the number of mentions for document type i, μ_i and σ_i are the mean and standard deviation of mentions across all biobanks for that document type, and Y is the biobank's age in years since its first mention. Each standardized score $\frac{r_i - \mu_i}{\sigma_i}$ is capped at $[-1, 1]$ to prevent outliers from dominating the metric. Since there are five document types, the total research impact R ranges from -5 to 5, where 5

represents exceptional visibility across all research sectors and -5 indicates minimal presence. Because mentions are standardized by document type, a biobank can have a negative value that results in a 'penalty' for its impact. The bIF scores higher on balanced biobanks, those having an impact, even if modest, across most sectors. Moreover, to minimize bias due to the length of time a biobank has been available, we normalize its bIF based on the number of years from the biobank's firs mention to 2023 (Y). The code to compute the research impact is found in `python/impact/cohort_impact_factor/impact_factor.py`.

The other factor that we consider for bIF is the scope and depth of research produced by each biobank. The disease impact D quantifies a biobank's contribution to disease research through three factors (see SI 7 for detailed definitions and equations): the breadth of conditions studied using the biobank, how deeply the biobank has contributed to research on specific conditions, and the biobank's contribution to rare disease research.

The biobank impact factor (bIF) of a biobank is a weighted sum of its research impact (M, Equation 0.8) and disease impact (D, Equation 0.1):

$$\text{bIF} = aM + bD \tag{0.9}$$

where a and b are the relative weights of each impact metric. We chose $a = 0.9$ and $b = 0.1$ given that they reflect the relative importance of the two impact scores, and research impact is a more general measure of the impact of a biobank across different biomedical sectors, while the other impact metric is specific to disease research (See SI 10.1 for weight sensitivity analysis that explores how different weightings affect the rankings of biobanks.).

The code for the bIF is at `python/impact/cohort_impact_factor/target.py`.

SI 10.1 Testing different weights to compute the bIF

In order to test our choice of parameters, we compared the rankings of ten biobanks across different (a, b) tuples representing the parameters of Equation 0.9 (Figure SI 10), namely (0.9, 0.1), (0.8, 0.2), and (0.7, 0.3). We found that from the top ten biobanks in terms of bIF, only two disappear from the top ten using other parameters, namely the European Collection of Cell Cultures (rank 11th with $a = 0.7$ and $b = 0.3$, and the Genotype-Tissue Expression Project (rank 12th with $a = 0.7$ and $b = 0.3$ and 11th with $a = 0.8$ and $b = 0.2$). In general, the rank distribution is robust across the three choices of parameters, with the Spearman correlation between the ranking obtained from our choice of parameters and those $a = 0.8$ and $b = 0.2$ being 0.99, and 0.97 when compared to those obtained using $a = 0.7$ and $b = 0.3$. Similarly, the results of the generalized linear model (SI 11) remain robust when using different parameters,

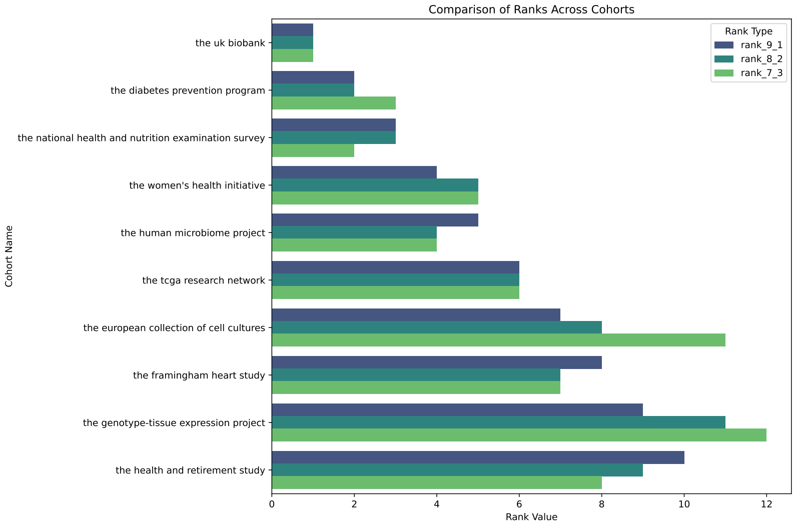

Figure SI 10: Biobank Impact Factor (bIF) for different parameters. The ranks of ten high-impact biobanks for three different choices of parameters (a, b) corresponding to the weights used to calculate the bIF.

with the only significance difference being that the biobank feature of having follow-up information becoming statistically significant after choosing different parameters (Figure SI 11).

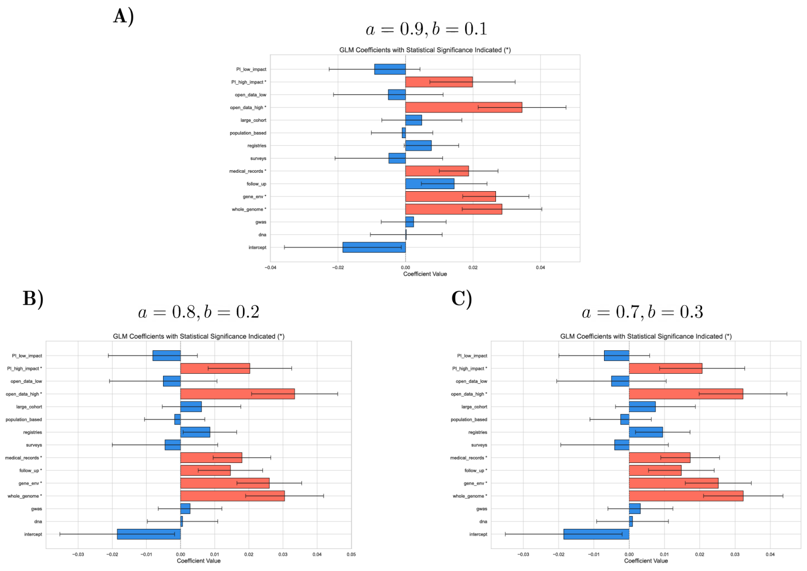

Figure SI 11: Model coefficients for different choices of weights a and b. The model used is described in SI 11.

SI 11 Generalized Linear Model

We used a Generalized Linear Model (GLM) to measure the relation between biobank data characteristics and bIF. The dependent variable is then bIF, and the 14 independent variables were all binary values depending on the data characteristics of each biobank (Tables SI 4 and SI 5). Specifically, we used a Gaussian family GLM with an identity function linked to the data using the `statsmodels` library in Python [34]. The model was applied to a set of 468 biobanks for which we could identify each feature (SI 9). For each dependent feature, we obtained its coefficient and associated p-value, labeling it as significant if p-value $0.05/n$, where n is the number of features in the model (Bonferroni correction for each independent feature). The model presents an R-square metric of 0.4 and a log-likelihood of 862.65. The code to fit the data to the GLM and to obtain the significant variables is at `python/stat_models/glm.py`.

The model's covariates include a large cohort sample (sample size in the top 10%), open data index (2 levels: low and high, SI 9.9), the biobank's PIs citation level at the time it was created (2 levels, low and high), population-based (1 for population-based biobanks, 0 for health-system based), available genetic data (sub-divided in GWAS, Whole-genome sequencing, and gene-environment data), registries, surveys & questionnaires, follow-up data, and medical records. The error term ϵ follows a standard normal distribution. It is important to note that bIF is a normalized metric of impact with a maximum of 0.47 for the UK Biobank, so possessing a feature with a coefficient of 0.01 sets a biobank in the top half of the bIF distribution.

Table SI 4: Summary description and statistics of the GLM model.

**Dep. Variable:**	target	**No. Observations:**	468
**Model:**	GLM	**Df Residuals:**	453
**Model Family:**	Gaussian	**Df Model:**	14
**Link Function:**	Identity	**Scale:**	0.0015157
**Method:**	IRLS	**Log-Likelihood:**	862.65
**Date:**	Mon, 15 Apr 2024	**Deviance:**	0.68663
**Time:**	10:02:51	**Pearson chi2:**	0.687
**No. Iterations:**	3	**Pseudo R-squ. (CS):**	0.4097
**Covariance Type:**	nonrobust		

Table SI 5: GLM features and their coefficients.

	**coef**	**std err**	**z**	**P> |z|**	**[0.025**	**0.975]**
**const**	-0.0186	0.009	-2.102	0.036	-0.036	-0.001
**dna**	0.0002	0.005	0.039	0.969	-0.010	0.011
**gwas**	0.0024	0.005	0.489	0.625	-0.007	0.012
**whole_genome**	0.0286	0.006	4.752	0.000	0.017	0.040
**gene_env**	0.0267	0.005	5.346	0.000	0.017	0.037
**follow_up**	0.0144	0.005	2.900	0.004	0.005	0.024
**medical_records**	0.0187	0.004	4.210	0.000	0.010	0.027
**surveys**	-0.0049	0.008	-0.608	0.543	-0.021	0.011
**registries**	0.0077	0.004	1.857	0.063	-0.000	0.016
**population**	-0.0010	0.005	-0.221	0.825	-0.010	0.008
**large_cohort**	0.0048	0.006	0.796	0.426	-0.007	0.017
**open_data_high**	0.0345	0.007	5.196	0.000	0.021	0.048
**open_data_low**	-0.0051	0.008	-0.618	0.537	-0.021	0.011
**PI_high_impact**	0.0198	0.006	3.079	0.002	0.007	0.032
**PI_low_impact**	-0.0092	0.007	-1.336	0.182	-0.023	0.004

SI 12 Code Availability

The code presented here can be found at https://github.com/Barabasi-Lab/quantifying_biobanks. The code and the accompanying data are also stored in a Zenodo repository at https://zenodo.org/records/11671294. A dashboard is available at http://biobanks.pythonanywhere.com/.

Bibliography

1. O'Donoghue, S., Dee, S., Byrne, J. A. & Watson, P. H. How Many Health Research Biobanks Are There? *Biopreservation and Biobanking.* Publisher: Mary Ann Liebert, Inc., publishers. ISSN: 1947-5535. https://www.liebertpub.com/doi/full/10.1089/bio.2021.0063 (2021) (Sept. 2021).
2. Inc., I. Population matters: Biobanks accelerate geno–pheno discoveries. en. Bandiera_abtest: a Cg_type: Advertisement Feature. https://www.nature.com/articles/d42473-020-00238-1 (2021).
3. Swertz, M. *et al.* Towards an Interoperable Ecosystem of Research Cohort and Real-world Data Catalogues Enabling Multi-center Studies. en. *Yearbook of Medical Informatics* **31.** Publisher: Georg Thieme Verlag KG, 262–272. ISSN: 0943-4747, 2364-0502. http://www.thieme-connect.de/DOI/DOI?10.1055/s-0042-1742522 (2024) (Aug. 2022).
4. Mabile, L. *et al.* Quantifying the use of bioresources for promoting their sharing in scientific research. *GigaScience* **2.** ISSN: 2047-217X. https://doi.org/10.1186/2047-217X-2-7 (2021) (Dec. 2013).
5. Annaratone, L. *et al.* Basic principles of biobanking: from biological samples to precision medicine for patients. en. *Virchows Archiv* **479,** 233–246. ISSN: 1432-2307. https://doi.org/10.1007/s00428-021-03151-0 (2024) (Aug. 2021).
6. Van Ommen, G.-J. B. *et al.* BBMRI-ERIC as a resource for pharmaceutical and life science industries: the development of biobank-based Expert Centres. en. *European Journal of Human Genetics* **23.** Publisher: Nature Publishing Group, 893–900. ISSN: 1476-5438. https://www.nature.com/articles/ejhg2014235 (2024) (July 2015).
7. Giffen, C. A. *et al.* Providing researchers with online access to NHLBI biospecimen collections: The results of the first six years of the NHLBI BioLINCC program. en. *PLOS ONE* **12.** Publisher: Public Library of Science, e0178141. ISSN: 1932-6203. https://journals.plos.org/plosone/article?id=10.1371/journal.pone.0178141 (2024) (June 2017).

8. Bose, N., Brookes, A. J., Scordis, P. & Visser, P. J. Data and sample sharing as an enabler for large-scale biomarker research and development: The EPND perspective. English. *Frontiers in Neurology* **13.** Publisher: Frontiers. ISSN: 1664-2295. https://www.frontiersin.org/journals/neurology/articles/10.3389/fneur.2022.1031091/full (2024) (Nov. 2022).

9. Liggins, C. *et al.* International Alzheimer's Disease Research Portfolio (IADRP) aims to capture global Alzheimer's disease research funding. en. *Alzheimer's & Dementia* **10.** _eprint: https://onlinelibrary.wiley.com/doi/pdf/10.1016/j.jalz.2013.12.013, 405–408. ISSN: 1552-5279. https://onlinelibrary.wiley.com/doi/abs/10.1016/j.jalz.2013.12.013 (2024) (2014).

10. Van der Velde, K. J. *et al.* MOLGENIS research: advanced bioinformatics data software for non-bioinformaticians. *Bioinformatics* **35,** 1076–1078. ISSN: 1367-4803. https://doi.org/10.1093/bioinformatics/bty742 (2024) (Mar. 2019).

11. Ouellette, S. & Tassé, A. M. P3G — 10 years of toolbuilding: From the population biobank to the clinic. *Applied & Translational Genomics* **3,** 36–40. ISSN: 2212-0661. https://www.ncbi.nlm.nih.gov/pmc/articles/PMC4882047/ (2024) (Apr. 2014).

12. Wang, Z. *et al.* Improved imputation of common and uncommon SNPs with a new reference set. en. *Nature Genetics* **44.** Publisher: Nature Publishing Group, 6–7. ISSN: 1546-1718. https://www.nature.com/articles/ng.1044 (2024) (Jan. 2012).

13. Bauermeister, S. *et al.* The Dementias Platform UK (DPUK) Data Portal. en. *European Journal of Epidemiology* **35,** 601–611. ISSN: 1573-7284. https://doi.org/10.1007/s10654-020-00633-4 (2024) (June 2020).

14. Vrijheid, M. *et al.* European Birth Cohorts for Environmental Health Research. *Environmental Health Perspectives* **120.** Publisher: Environmental Health Perspectives, 29–37. https://ehp.niehs.nih.gov/doi/full/10.1289/ehp.1103823 (2024) (Jan. 2012).

15. Kennedy, A. E. *et al.* The Cancer Epidemiology Descriptive Cohort Database: A Tool to Support Population-Based Interdisciplinary Research. *Cancer Epidemiology, Biomarkers & Prevention* **25,** 1392–1401. ISSN: 1055-9965. https://doi.org/10.1158/1055-9965.EPI-16-0412 (2024) (Oct. 2016).

16. Smith-Warner, S. A. *et al.* Methods for Pooling Results of Epidemiologic Studies: The Pooling Project of Prospective Studies of Diet and Cancer. *American Journal of Epidemiology* **163,** 1053–1064. ISSN: 0002-9262. https://doi.org/10.1093/aje/kwj127 (2024) (June 2006).

17. Bandrowski, A. A decade of GigaScience: What can be learned from half a million RRIDs in the scientific literature? *GigaScience* **11,** giac058. ISSN: 2047-217X. https://doi.org/10.1093/gigascience/giac058 (2024) (Jan. 2022).

18. Bergeron, J., Doiron, D., Marcon, Y., Ferretti, V. & Fortier, I. Fostering population-based cohort data discovery: The Maelstrom Research cataloguing toolkit. en. *PLOS ONE* **13.** Publisher: Public Library of Science, e0200926. ISSN: 1932-6203. https://journals.plos.org/plosone/article?id=10.1371/journal.pone.0200926 (2024) (July 2018).

19. Tryka, K. A. *et al.* NCBI's Database of Genotypes and Phenotypes: dbGaP. *Nucleic Acids Research* **42,** D975–D979. ISSN: 0305-1048. https://doi.org/10.1093/nar/gkt1211 (2024) (Jan. 2014).

20. Lerche, S. *et al.* Methods in Neuroepidemiology Characterization of European Longitudinal Cohort Studies in Parkinson's Disease - Report of the JPND Working Group BioLoC-PD. *Neuroepidemiology* **45,** 282–297. ISSN: 0251-5350. https://doi.org/10.1159/000439221 (2024) (Nov. 2015).

21. Sollis, E. *et al.* The NHGRI-EBI GWAS Catalog: knowledgebase and deposition resource. *Nucleic Acids Research* **51,** D977–D985. ISSN: 0305-1048. https://doi.org/10.1093/nar/gkac1010 (2024) (Jan. 2023).

22. Wijmenga, C. & Zhernakova, A. The importance of cohort studies in the post-GWAS era. en. *Nature Genetics* **50.** Publisher: Nature Publishing Group, 322–328. ISSN: 1546-1718. https://www.nature.com/articles/s41588-018-0066-3 (2024) (Mar. 2018).

23. Yan, E. & Ding, Y. Scholarly network similarities: How bibliographic coupling networks, citation networks, cocitation networks, topical networks, coauthorship networks, and coword networks relate to each other. en. *Journal of the American Society for Information Science and Technology* **63.** _eprint: https://onlinelibrary.wiley.com/doi/ 1313–1326. ISSN: 1532-2890. https://onlinelibrary.wiley.com/doi/abs/10.1002/asi.22680 (2025) (2012).

24. Blondel, V. D., Guillaume, J.-L., Lambiotte, R. & Lefebvre, E. Fast unfolding of communities in large networks. en. *Journal of Statistical Mechanics: Theory and Experiment* **2008,** P10008. ISSN: 1742-5468. https://dx.doi.org/10.1088/1742-5468/2008/10/P10008 (2025) (Oct. 2008).

25. Lancichinetti, A. & Fortunato, S. Limits of modularity maximization in community detection. *Physical Review E* **84.** Publisher: American Physical Society, 066122. https://link.aps.org/doi/10.1103/PhysRevE.84.066122 (2025) (Dec. 2011).

26. Newman, M. E. J. & Girvan, M. Finding and evaluating community structure in networks. *Physical Review E* **69.** Publisher: American Physical Society, 026113. https://link.aps.org/doi/10.1103/PhysRevE.69.026113 (2025) (Feb. 2004).

27. Fortunato, S. & Hric, D. Community detection in networks: A user guide. *Physics Reports. Community detection in networks: A user guide* **659,** 1–44. ISSN: 0370-1573. https://www.sciencedirect.com/science/article/pii/S0370157316302964 (2025) (Nov. 2016).

28. Lancichinetti, A., Fortunato, S. & Kertész, J. Detecting the overlapping and hierarchical community structure in complex networks. en. *New Journal of Physics* **11,** 033015. ISSN: 1367-2630. https://dx.doi.org/10.1088/1367-2630/11/3/033015 (2025) (Mar. 2009).

29. Hric, D., Darst, R. K. & Fortunato, S. Community detection in networks: Structural communities versus ground truth. *Physical Review E* **90.** Publisher: American Physical Society, 062805. https://link.aps.org/doi/10.1103/PhysRevE.90.062805 (2025) (Dec. 2014).

30. Meng, X., Varol, O. & Barabási, A.-L. Hidden citations obscure true impact in science. *PNAS Nexus* **3,** pgae155. ISSN: 2752-6542. https://doi.org/10.1093/pnasnexus/pgae155 (2024) (May 2024).

31. Cabanac, G. Extracting and quantifying eponyms in full-text articles. en. *Scientometrics* **98,** 1631–1645. ISSN: 1588-2861. https://doi.org/10.1007/s11192-013-1091-8 (2024) (Mar. 2014).

32. McCain, K. W. Eponymy and Obliteration by Incorporation: The case of the "Nash Equilibrium". en. *Journal of the American Society for Information Science and Technology* **62.** _eprint: https://onlinelibrary.wiley.com/doi/pdf/10.1002/asi.21536, 1412–1424. ISSN: 1532-2890. https://onlinelibrary.wiley.com/doi/abs/10.1002/asi.21536 (2024) (2011).

33. Baker, M. Building better biobanks. en. *Nature* **486.** Bandiera_abtest: a Cg_type: Nature Research Journals Number: 7401 Primary_atype: Special Features Publisher: Nature Publishing Group Subject_term: Diseases;Molecular biology Subject_term_id: diseases;molecular-biology, 141–146. ISSN: 1476-4687. https://www.nature.com/articles/486141a (2021) (June 2012).

34. Seabold, S. & Perktold, J. *Statsmodels: Econometric and Statistical Modeling with Python* en. in (Austin, Texas, 2010), 92–96. https://doi.curvenote.com/10.25080/Majora-92bf1922-011 (2025).
